# A straightforward approach to antibodies recognising cancer specific glycopeptidic neoepitopes†

**DOI:** 10.1039/d0sc00317d

**Published:** 2020-04-30

**Authors:** Hajime Wakui, Yoshikazu Tanaka, Toyoyuki Ose, Isamu Matsumoto, Koji Kato, Yao Min, Taro Tachibana, Masaharu Sato, Kentaro Naruchi, Fayna Garcia Martin, Hiroshi Hinou, Shin-Ichiro Nishimura

**Affiliations:** Field of Drug Discovery Research, Faculty of Advanced Life Science, Graduate School of Life Science, Hokkaido University N21 W11, Kita-ku Sapporo 001-0021 Japan shin@sci.hokudai.ac.jp; Graduate School of Life Sciences, Tohoku University 2-1-1 Katahira, Aoba-ku Sendai 980-8577 Japan; Field of X-ray Structural Biology, Faculty of Advanced Life Science, Graduate School of Life Science, Hokkaido University N10 W8, Kita-ku Sapporo 060-0810 Japan; Research Institute for Interdisciplinary Science and Graduate School of Natural Science and Technology, Okayama University 3-1-1, Tsushima-naka, Kita-ku Okayama 700-8530 Japan; Department of Bioengineering, Graduate School of Engineering, Osaka City University Sumiyoshi-ku Osaka 558-8585 Japan; Medicinal Chemistry Pharmaceuticals, Co., Ltd. N9 W15, Chuo-ku Sapporo 060-0009 Japan

## Abstract

Aberrantly truncated immature *O*-glycosylation in proteins occurs in essentially all types of epithelial cancer cells, which was demonstrated to be a common feature of most adenocarcinomas and strongly associated with cancer proliferation and metastasis. Although extensive efforts have been made toward the development of anticancer antibodies targeting MUC1, one of the most studied mucins having cancer-relevant immature *O*-glycans, no anti-MUC1 antibody recognises carbohydrates and the proximal MUC1 peptide region, concurrently. Here we present a general strategy that allows for the creation of antibodies interacting specifically with glycopeptidic neoepitopes by using homogeneous synthetic MUC1 glycopeptides designed for the streamlined process of immunization, antibody screening, three-dimensional structure analysis, epitope mapping and biochemical analysis. The X-ray crystal structure of the anti-MUC1 monoclonal antibody SN-101 complexed with the antigenic glycopeptide provides for the first time evidence that SN-101 recognises specifically the essential epitope by forming multiple hydrogen bonds both with the proximal peptide and GalNAc linked to the threonine residue, concurrently. Remarkably, the structure of the MUC1 glycopeptide in complex with SN-101 is identical to its solution NMR structure, an extended conformation induced by site-specific glycosylation. We demonstrate that this method accelerates dramatically the development of a new class of designated antibodies targeting a variety of “dynamic neoepitopes” elaborated by disease-specific *O*-glycosylation in the immunodominant mucin domains and mucin-like sequences found in intrinsically disordered regions of many proteins.

## Abstract 2

We developed new class of designated antibodies targeting of “dynamic neoepitopes” elaborated by disease-specific *O*-glycosylation at the immunodominant mucin domains.

## Graphical abstract

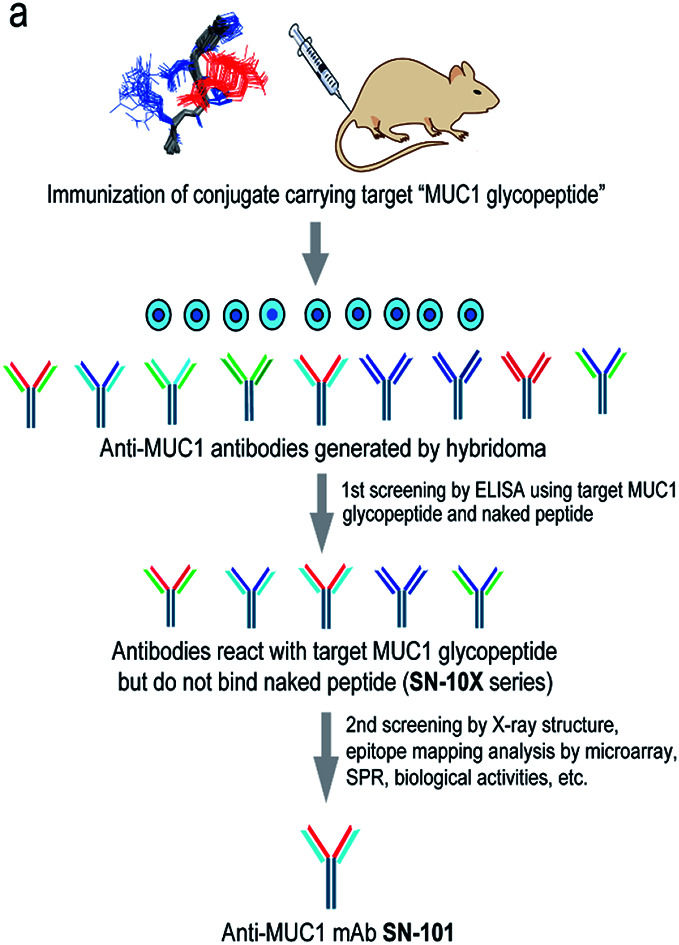

## Introduction

As a pilot study conducted by the National Cancer Institute (NCI), the Translational Research Working Group ranked MUC1 as the second best potential target out of 75 tumour-associated antigens,^1^ and extensive efforts have been made toward the development of anticancer antibodies and vaccines targeting cancer cell-derived MUC1 glycoproteins.^2^ Cancer cells express a high level of MUC1 modified with immature truncated *O*-glycans such as Tn (GalNAcα1→), T (Galβ1 → 3GalNAcα1→), STn (Neu5Acα2 → 6GalNAcα1→), and ST (Neu5Acα2 → 3Galβ1 → 3GalNAcα1→) antigens.^2,3^ The emerging importance of aberrantly glycosylated MUC1 in cancer proliferation and metastasis^4–6^ has motivated us to develop antibodies targeting MUC1 extracellular tandem repeats bearing these key glycoforms.^7–9^ The Tn antigen is the simplest and one of the most important structural motifs found widely in various aggressive carcinomas.^3,10^ Despite extensive efforts for the development of antibodies to MUC1 having the Tn antigen, it is demonstrated that most anti-MUC1 antibodies do not interact directly with carbohydrates while binding affinities with the immunodominant MUC1 peptides appear to be enhanced significantly by *O*-glycosylation in this area.^8,11,12^ Indeed, recent X-ray crystallographic studies on the structures of anti-MUC1 monoclonal antibodies (mAbs) such as SM3 (ref. 13) and AR20.5 (ref. 14) complexed with synthetic MUC1 glycopeptides demonstrated that carbohydrates do not form any specific polar contacts with these mAbs.

In contrast, we demonstrated that anti-MUC1/KL6 mAb, an antibody probing MUC1/KL6 fragments as a sensitive serum biomarker of interstitial lung diseases (ILDs),^15^ recognises specifically the trisaccharide ST antigen attached to the Thr residue of a heptapeptide (Pro-Asp-Thr-Arg-Pro-Ala-Pro) epitope within MUC1 tandem repeats.^16^ Strikingly, anti-MUC1/KL6 mAb discriminates the ST antigen from Tn and T antigens at the same *O*-glycosylation site independent of the modifications at other potential sites.^11,12,16^ NMR study uncovers that sialylation of the T antigen at the *O*-3 position of Gal residue induces conformational alteration of this immunodominant peptide region from β-turn like *cis* to an extended *trans* structure.^17^ These results suggest that anti-MUC1/KL6 mAb recognises strictly a glycopeptidic epitope, an extended *trans* conformation of this peptide motif modified specifically with the ST antigen. However, it is also revealed that sugar extensions at the *O*-6 position of GalNAc residue at the reducing end of this minimal epitope do not impede the interaction of MUC1 with this antibody.^12,16^ This means that anti-MUC1/KL6 mAb reacts with MUC1 fragments bearing core 2 type ST variants and disialyl T (dST) found widely in normal tissues. These results indicate that anti-MUC1/KL6 mAb often shows a high level of reactivities with sera of healthy control and many cancer patients^18^ in addition to ILDs.^15^ It should be emphasized that lack of carbohydrate-binding specificities in most anti-MUC1 mAbs makes the development of MUC1-based therapeutic antibodies difficult while Clausen *et al.* obtained anti-MUC1 mAbs (5 × 10^5^ and 2D9) interacting GSTA with Tn or STn moieties.^19^ Antibodies that bind cancer-relevant glycopeptidic neoepitopes with much higher specificities in carbohydrate recognition will provide dramatic improvements in the therapeutic and diagnostic index of anti-MUC1 mAbs toward clinical translation to patient care.

Our attention has been paid to the development of a new class of antibodies exhibiting ability to recognise glycopeptidic neoepitopes by interacting directly with both carbohydrate epitopes and the proximal peptide sequence, concurrently. The SN-101 antibody, one of the potential anti-MUC1 mAbs established by a novel strategy, recognises specifically a targeted Tn-glycosylated MUC1 peptide. Here, we present the first three-dimensional structure of anti-MUC1 mAb (SN-101) that interacts with MUC1 fragments through multiple polar contacts both with the carbohydrate side chain and the proximal immunodominant peptide region. The use of the synthetic glycopeptide library allows for the development of a new class of antibodies targeting “dynamic glycopeptidic neoepitopes” elaborated by disease-relevant *O*-glycosylation in immunodominant mucin domains.

## Results

### Generation of antibodies targeting glycopeptidic epitopes

**Fig. 1:**
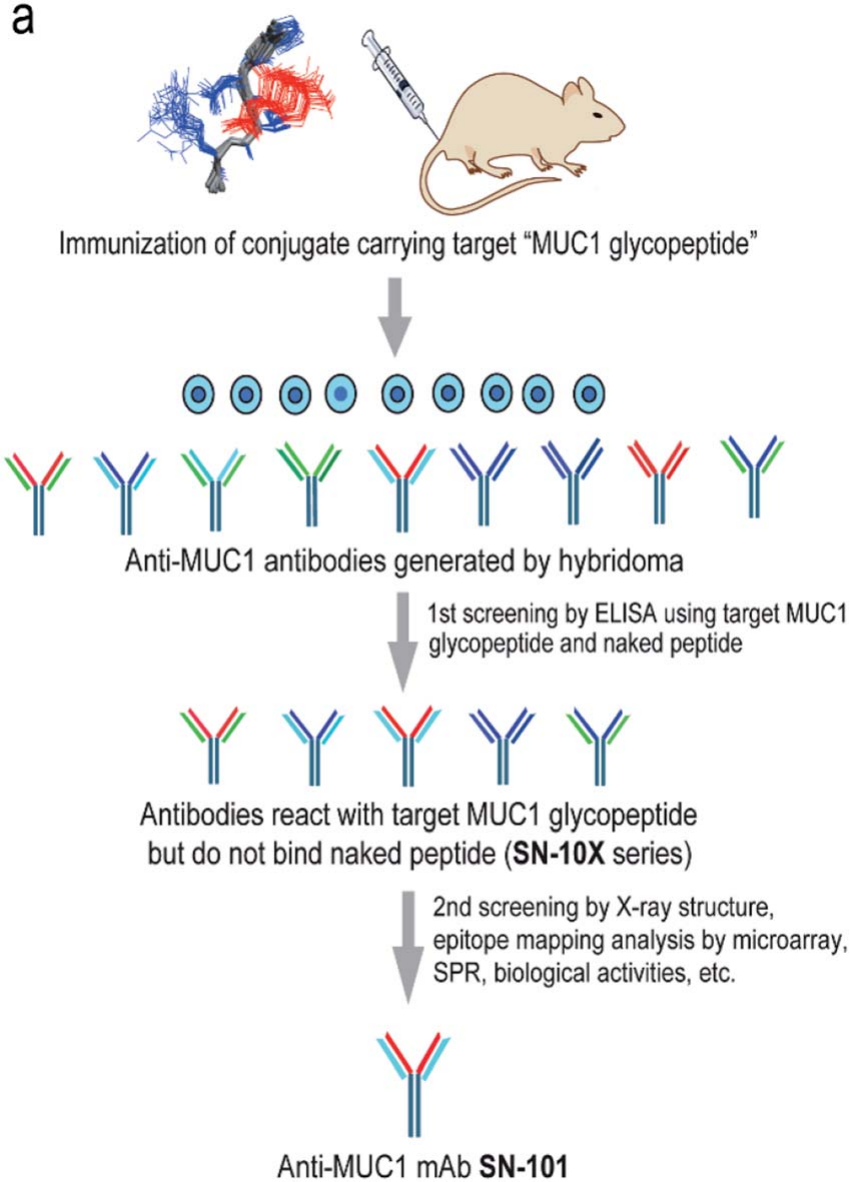
Generation of epitope-defined anti-MUC1 antibodies. (a) A strategy for the generation of antibodies targeting glycopeptidic epitopes by using synthetic glycopeptides designed for the streamlined process from the immunization of “conformational glycopeptidic neoepitopes”, antibody selection, and characterization. (b) A list of compounds used in this study. Compound **1** was conjugated with KLH by using the Cys residue (red) or aminooxy-functionalized nanoparticles^25–27^ by using the ketone linker (blue) and used for the immunization. The first screening was performed by ELISA immobilizing compounds **1** and **2** using Cys residue (red) to collect antibodies binding selectively with glycopeptide **1**. Compound **3** was used for the co-crystallization with SN-101. Compounds **4–24** were displayed on the microarray by means of the ketone linker (blue) and employed for epitope mapping analysis. Compound **25** was used for the SPR analysis by immobilizing with Cys residue (red).

Fig. 1a shows a strategy for the generation of antibodies targeting “glycopeptidic epitopes” by means of homogeneous synthetic glycopeptides as key materials. MUC1 glycopeptide/peptide derivatives **1–25** were designed for the development of antibodies recognizing MUC1 having the Tn antigen at the immunodominant Asp-Thr-Arg motif and synthesized efficiently according to the method reported previously (Fig. 1b).^11,12,16,20–23^ To obtain antibodies recognising the MUC1 glycopeptide containing Tn-glycosylated immunodominant motif, compound **1** conjugated with keyhole limpet hemocyanin (KLH) was immunized at the tail base of BDF-1 mice and the collected iliac lymph node lymphocytes were fused with myeloma SP2 cells.^24^ The antibody-producing hybridomas were screened by using an Enzyme-Linked Immunosorbent Assay (ELISA) plate immobilizing compounds **1** and **2** to find whether antibodies have the ability to discriminate the target MUC1 glycopeptide **1** from non-glycosylated MUC1 peptide **2**. Next, antibodies reacting with MUC1 glycopeptide **1** but not with naked MUC1 peptide **2** denoted as SN-10X antibodies were subjected to systematic characterization such as X-ray crystallographic structural assessment by means of an epitope model MUC1 glycopeptide **3** as a ligand, epitope mapping analysis by using microarray displaying compounds **4–24**, SPR analysis using a sensor chip immobilizing compound **25**, and further various biochemical tests. Consequently, SN-101 (IgG1) is the first antibody among anti-MUC1 mAbs selectively recognising cancer-relevant Tn-glycosylated MUC1 fragments screened by our approach that enables precise analyses of the crystal structures of Fab and its complex with a target MUC1 glycopeptidic neoepitope (ESI Fig. S1†).

### Crystal structures of SN-101 and its complex with Tn-MUC1 glycopeptide

**Fig. 2:**
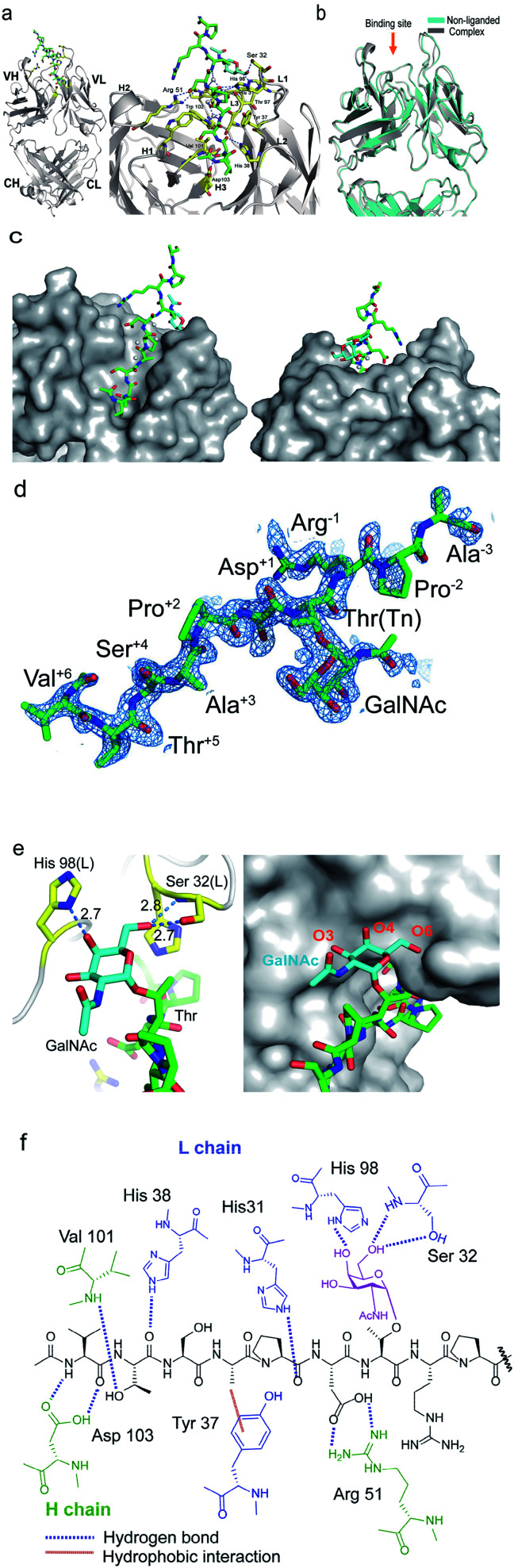
Crystal structures of SN-101 Fab and its complex with the MUC1 glycopeptide **3** (PDB: 6KX0 and 6KX1). (a) Overall structure (left) and an enlarged picture focusing on the binding site (right) of SN-101 Fab complexed with **3**. (b) Superposition of two SN-101 Fab structures of the complex (blue) and non-liganded (gray) forms. (c) Surface representations of SN-101 in complex with **3** (gray), seen from the back and front. Glycopeptide **3** is shown as a stick model with carbon atoms in green. (d) 2*F*_o_ − *F*_c_ electron density map represents 10 amino acid residues of 15 mer MUC1 glycopeptide **3** bound to SN-101. (e) 3D structures focusing on GalNAc recognition by SN-101. Binding interaction is mediated by hydrogen bonds between *O*-4 and *O*-6 of GalNAc (blue) with His98 and Ser32 of the light chain (yellow), respectively (left). SN-101 surface represents the topology of the GalNAc binding site (left). (f) Schematic image of the interactions between SN-101 Fab and MUC1 glycopeptide **3**.

To decipher the structural basis in the recognition of Tn-MUC1 glycopeptide by the SN-101 antibody at the atomic level, a pure Fab fragment of SN-101 was crystalized in the presence and absence of synthetic MUC1 glycopeptide **3**, Ac-Val-Thr-Ser-Ala-Pro-Asp-Thr(Tn)-Arg-Pro-Ala-Pro-Gly-Ser-Thr-Ala-NH2. The results of our previous study provided evidence that compound **3** includes the common epitope region Ala-Pro-Asp-Thr-Arg-Pro-Ala-Pro recognized by most anti-MUC1 antibodies.^11,16^ The crystals of SN-101 complexed with MUC1 glycopeptide **3** and non-liganded enabled structural analysis at 1.77 Å and 2.40 Å, respectively (ESI Table S1†). Fig. 2a shows the overall structure of SN-101 Fab complexed with MUC1 glycopeptide **3** and an enlarged view focusing on the binding site. The overall structures of complexed and non-liganded crystals were almost identical in the main and side chains, and the RMSD value between the two structures was 0.63 Å (Fig. 2b).

The glycopeptide could be confirmed at the CDRs of the surface groove of the SN-101 Fab (Fig. 2c) and assigned to Val-Thr-Ser-Ala-Pro-Asp-Thr(Tn)-Arg-Pro-Ala-Pro-Gly-Ser-Thr-Ala from the electron density (Fig. 2d). Strikingly, the crystallographic analysis uncovered that SN-101 forms hydrogen bonds with *O*-4 and *O*-6 of GalNAc residue of the MUC1 glycopeptide **3** by using His98L and Ser32L residues. It was also demonstrated that Val, Thr, Pro, and Asp residues of the proximal peptide region are involved in hydrogen bonding with Asp103H, Val101H, His38L, Asp103H, His31L, and Arg51H as summarised in Table 1. The side chain of Ala is engaged in hydrophobic contact with Tyr37L. Interestingly, Thr and Ser of the Val-Thr-Ser-Ala motif in the N-terminal side are inserted deeply into the binding pocket of SN-101, indicating that additional *O*-glycosylation in the Val-Thr-Ser-Ala region may inhibit the binding of SN-101 with Tn-glycosylated MUC1 fragments. Most importantly, structural features provide evidence that SN-101 recognizes specifically the GalNAc moiety (Tn antigen) and the immunodominant peptide motif, concurrently. Furthermore, there is little space for the interaction of SN-101 with longer and mature *O*-glycans generated by sugar extension at both the C-3 and C-6 positions of GalNAc residue of the MUC1 glycopeptide (Fig. 2e), suggesting that SN-101 can discriminate the Tn antigen from other glycans such as T, ST, and STn antigens, and core 2 type *O*-glycans. Fig. 2f shows the schematic image of the polar contacts and hydrophobic interaction between SN-101 Fab and the MUC1 glycopeptide **3**.

**Table 1:** Hydrogen bonds between SN-101 and MUC1 glycopeptide **3**

Glycopeptide	SN-101 Fab	Hydrogen bond	Distance (Å)
Val	Asp103H	N–O_*δ*_2	2.94
Thr	Val101H	O_*γ*_–N	2.92
	His38L	O–N_*ε*_	3.20
	Asp103H	O_*γ*_–O_*δ*_1	2.50
Pro	His31L	N_*ε*_–O	2.73
Asp	Arg51H	O_*δ*_2–N_*η*_1	2.73
		O_*δ*_1–N_*η*_2	3.09
GalNAc	Ser32L	O6–O_*γ*_	2.63
		O6–N	2.84
	His98L	O4–N_*δ*_1	2.70

### SN-101 recognises specifically a dynamic glycopeptidic neoepitope

**Fig. 3:**
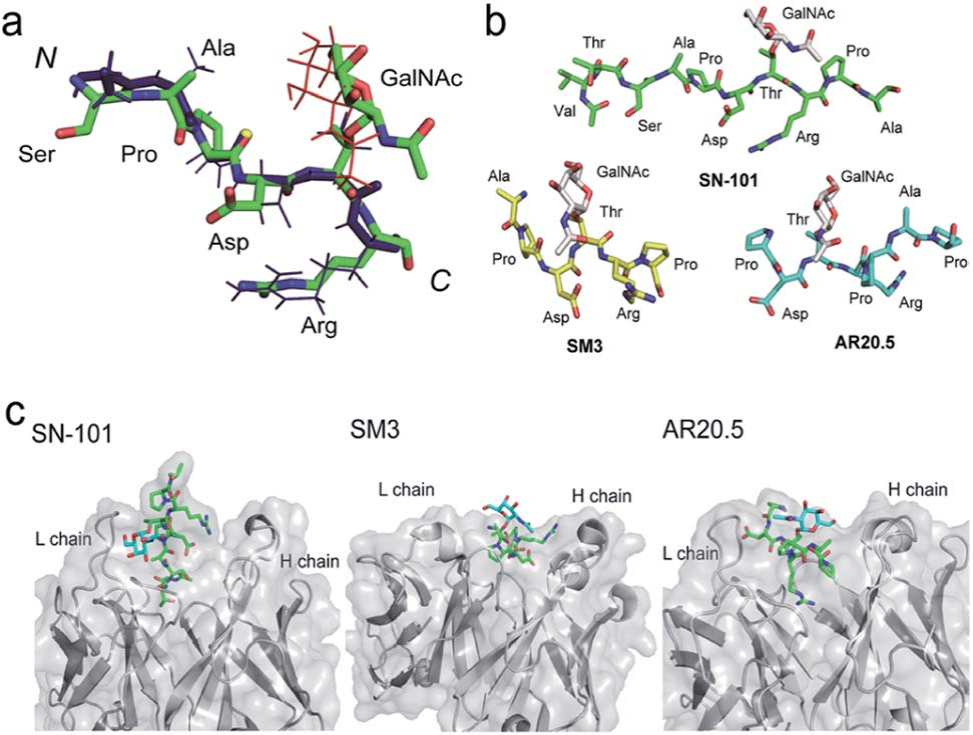
SN-101 recognises the specifically dynamic glycopeptidic neoepitope. (a) Superposition of the solution NMR structure of 23 mer MUC1 glycopeptide [Ac-Gly-Val-Thr-Ser-Ala-Pro-Asp-Thr(Tn)-Arg-Pro-Ala-Pro-Gly-Ser-Thr-Ala-Pro-Pro-His-Gly-Val-Thr-NH2]^12^ and the X-ray crystal structure of 15 mer MUC1 glycopeptide **3** complexed with SN-101. 30 lowest energy NMR structures in the Ser-Ala-Pro-Asp-Thr(Tn)-Arg region was overlaid. Blue stick represents the peptide backbone of the NMR structure. (b) Conformations of Tn-glycosylated MUC fragments bound to SN-101, SM3,^13^ and AR20.5.^14^ (c) Comparison of the X-ray crystal structures of SN-101 (this study, PDB: 6KX1), SM3 (PDB: 5A2K), and AR20.5 (PDB: 5T78) in complex with Tn-glycosylated MUC1 fragments.

We demonstrated that SN-101 interacts directly with both the GalNAc and peptide portions of Tn-glycosylated MUC1 fragment **3** through specific polar contacts (Fig. 2 and Table 1). However, the effect of the site-specific glycosylation-induced conformational alteration observed in the MUC1 fragments on the molecular mechanism in antibody recognition remains elusive.^8,14,17,28–31^ To assess whether SN-101 recognizes a conformational epitope generated dynamically by Tn-glycosylation of MUC1 in solution, we compared the X-ray crystal structure of MUC1 glycopeptide **3** bound to SN-101 with the solution NMR structure of the synthetic MUC1 glycopeptide having a Tn antigen at the immunodominant Asp-Thr-Arg motif^12^ (Fig. 3a and Table 2). Surprisingly, it was revealed that the two structures are mostly identical and the RMSD value in the peptide backbone between the solution NMR structure and X-ray crystal structure within SN-101 is estimated to be 0.67 Å. The result clearly demonstrates that SN-101 recognizes the specifically conformational glycopeptidic neoepitope elaborated dynamically in solution by Tn-glycosylation in this peptide region. As shown in Fig. 3b, it is likely that the conformations of the MUC1 glycopeptides in complex with three antibodies could be denoted as a sort of extended structures when compared with a type I β-turn structure of the 9 mer non-glycosylated MUC1 peptide found by solution NMR study.^30^ However, the present results uncover that Tn-glycosylated MUC1 fragments identified within the complexes with SM3 and AR20.5 form apparently different conformations from that determined in the complex with SN-101 (Fig. 3b), in which the conformation of the bound MUC1 glycopeptide **3** is completely merged with that of its solution NMR structure (Fig. 3a). Notably, SN-101 recognizes the solution structure of this glycopeptidic neoepitope by interacting both with the GalNAc moiety and the immunodominant peptide, concurrently. In contrast, although both SM3 and AR20.5 bind dominantly with the peptide backbone of the MUC1 fragments, they do not directly interact with GalNAc residue (Fig. 3c).^13,14,28^ These observations clearly demonstrate that the structural basis in the recognition of Tn-glycosylated MUC1 fragments by SN-101 differs entirely from those by SM3 and AR20.5.

**Table 2:** Dihedral angle of glycopeptide **3** in crystals and solution^a^

Residue	*ϕ* (°)		*ψ* (°)	
	X-ray	NMR	X-ray	NMR
Ser	−82.9	−117.1	134.0	172.1
Ala	−98.1	−100.5	149.1	147.7
Pro	−68.0	−53.0	138.0	157.0
Asp	−74.3	−79.7	151.4	163.6
Thr	−110.2	−124.7	133.7	71.7
Arg	−141.6	−79.5	70.7	164.3

a The conformation of the glycoside bond (X-ray): *ϕ* (°) = O5–C1–O–Cβ = 88.4 and *ψ* (°) = C1–O–Cβ–Cα = 147.1; (NMR): *ϕ* (°) = O5–C1–O–Cβ = 59.2 and *ψ* (°) = C1–O–Cβ–Cα = 157.8.

### SN-101 binds with Tn-glycosylated MUC1 but not with T-glycosylated MUC1

**Fig. 4:**
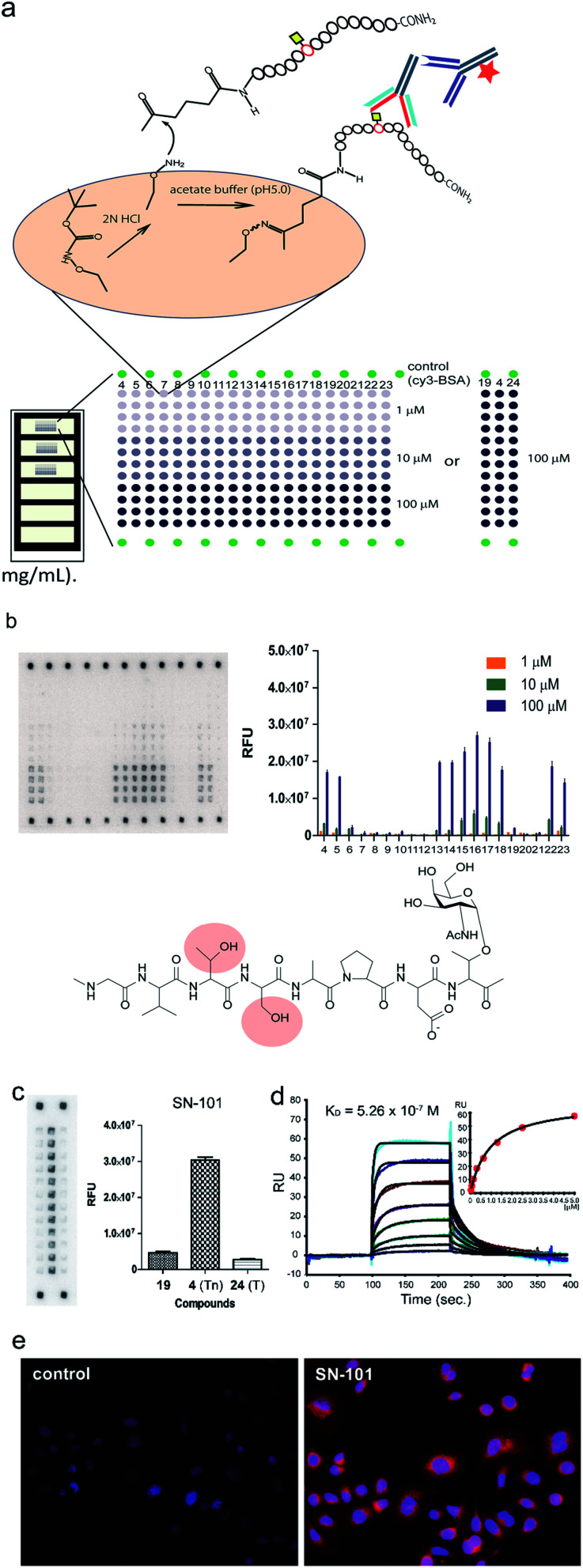
Interaction of SN-101 with synthetic MUC1 glycopeptides and membrane bound MUC1 of human breast cancer cells. (a) Preparation and layout of the microarray displaying MUC1 peptide and glycopeptides. (b) Epitope mapping analysis of SN-101 using a microarray displaying compounds **4–24**. Compounds **4–12** are glycopeptides whose N-terminal residues decrease one by one. Similarly, compounds **13–18** are the glycopeptides whose C-terminal residues increase one by one. A naked MUC1 peptide **19**, MUC1 fragments having two Tn antigens **20–23**, and compound **24** with the T antigen at immunodominant Pro-Asp-Thr-Arg are also used. The results represent the average fluorescence intensities of the spots (*n* = 4) for all compounds in each concentration (1, 10, and 100 μM). The chemical structure shows an essential epitope for SN-101 and glycosylation at the highlighted Thr or Ser residue abrogates the antibody recognition. (c) Glycan specific binding of SN-101 revealed by glycoform-focused microarray prepared from 100 μM MUC1 glycopeptides **4**, **19**, and **24**. The results represent the average fluorescence intensities of spots (*n* = 12) for compounds **19** (naked), **4** (Tn antigen), and **24** (T antigen), respectively. (d) Binding affinity of SN-101 Fab to a MUC1 glycopeptide **25** determined from the SPR binding curve (*K*_D_ = 5.26 × 10^−7^ M). (e) Interaction of SN-101 with cancer cell surface MUC1 illuminated by fluorescence-labelled anti-mouse IgG mAb.

To determine an essential epitope of SN-101, we assessed the binding specificity of this antibody for compounds **4–24** displayed on the microarray prepared according to the methods reported previously.^11,12^ As shown in Fig. 4a, synthetic MUC1 fragments modified with a reactive ketone linker at the N-terminus were immobilized directly on the microarray surface through oxime bond formation with the aminooxy-functional groups of the coated antifouling copolymer. The antibody bound to glycopeptides displayed on the microarray can be detected by probing with a fluorescence (Cy3)-labelled goat anti-mouse IgG antibody (Fig. 4b). The results showed that SN-101 binds preferentially with an essential/minimal epitope Gly-Val-Thr-Ser-Pro-Asp-Thr(Tn) within MUC1 tandem repeats. Intriguingly, SN-101 cannot bind with compounds **20** and **21**, indicating that the glycosylation of the neighbouring Thr and Ser residues involved in the Val-Thr-Ser-Ala region of the N-terminal side impedes antibody recognition of this epitope as highlighted in the minimal epitope structure shown in Fig. 4b. In contrast, modifications of Ser (compound **22**) and/or Thr (compound **23**) residues located in the C-terminal side do not affect the antibody recognition. It is interesting to note that the conformational impact of glycosylation at Val-Thr-Ser-Ala on the Pro-Asp-Thr-Arg may influence the antibody binding.^12^ Most importantly, it was demonstrated that SN-101 discriminates the difference between Tn-glycosylated MUC1 peptide **4** and T-glycosylated variant **24** (Fig. 4c). To our knowledge, none of the anti-MUC1 mAbs shows such Tn-glycoform specific recognition in this immunodominant region of MUC1 tandem repeats.^8,9,11,12,32^ Intriguingly, these results of epitope mapping analysis could be mostly predicted using the X-ray crystal structure of SN-101 in complex with MUC1 glycopeptide **3**. As indicated in Fig. 2c and e, the X-ray structures clearly uncovered that (a) the N-terminal Val-Thr-Ser-Ala region of MUC1 fragment 3 is inserted deeply into the binding cavity of SN-101 and (b) SN-101 binds specifically GalNAc residue through the critical hydrogen bonds with the *O*-4 and *O*-6 atoms of hydroxyl groups by using His98L and Ser32L residues. These characteristic features in the combined structure clearly indicate the abovementioned restrictions in the antibody recognition. The dissociation constant of SN-101 Fab with MUC1 glycopeptide **25** was estimated to be *K*_D_ = 5.26 × 10^−7^ M by surface plasmon resonance (SPR) measurements (Fig. 4d), indicating that the strength of affinity appears to be lower than that for other anti-MUC1 antibodies^32^ but further engineering to improve binding affinity with this target epitope is under way. For example, the addition of ability to interact with the *N*-acetyl group of GalNAc will enhance the affinity of the antibody. However, a preliminary experiment revealed that SN-101 interacts with native MUC1 expressed on the surface of cultured cancer cells, HER2 negative human breast cancer OCUB-M cells^33^ (Fig. 4e), whereas SN-101 shows a weak inhibitory effect on the proliferation of OCUB-M cells (IC_50_ = 0.6 mg mL^−1^).

## Discussion

### MUC1 glycopeptidic neoepitopes are highly potential molecular targets

The circulation of MUC1 fragments shed from a cancer cell surface is used as a biomarker for diagnosing cancer stages and monitoring relapse after treatments.^34,35^ Importantly, it was reported that extracellular MUC1 tandem repeats of cancer cells modulate the tumour microenvironment through interaction with some pivotal lectins in a glycoform-dependent manner.^36–41^ Our recent results also uncovered that the binding mode of *O*-glycans attached to MUC1 fragments with lectins such as galectins^42^ and macrophage galactose-type lectin (MGL)^43^ is often affected by the characteristic features of the proximal peptide regions. Although MUC1 is a promising molecular target for cancer therapy, our understanding of the biosynthetic mechanisms as well as functional roles of MUC1 modified with many important *O*-glycans in cancer biology is still highly limited.^2,44^ Accumulated evidence indicates that the broad binding specificities of most known anti-MUC1 mAbs toward *O*-glycans of MUC1 fragments make precise and accurate diagnosis difficult.^11,12,15,18,32^ Therefore, the advent of antibodies having the ability to recognise precisely MUC1 fragments bearing immature truncated glycoforms both with glycan and peptide sequence specificities has been strongly required because such antibodies can greatly contribute to insights into the biological significance of glycosylation in MUC1 and development of novel anticancer therapeutic and diagnostic antibodies.

### SN-101 recognizes a conformational glycopeptidic neoepitope

Here we established a rational and straightforward strategy for the generation of antibodies reacting specifically with designated glycopeptide structures (Fig. 1), namely a novel class of “glycopeptide epitope-defined antibodies”. The use of the tailored compound library of homogeneous synthetic peptide/glycopeptides allows for the seamless scheme of immunisation of a targeted glycopeptide, antibody screening, and systematic structural and biochemical characterisation of selected antibodies toward the development of glycopeptide epitope-defined antibodies. Our results provide evidence that (a) the X-ray crystal structure of this glycopeptidic neoepitope in complex with SN-101 is well converged with its solution NMR structure, (b) SN-101 recognises this glycopeptidic neoepitope by interacting concurrently with both the GalNAc moiety and the immunodominant peptide region, and (c) SN-101 interacts with Tn-glycosylated MUC1 but not with T-glycosylated MUC1 fragments. These results clearly demonstrate that structural characteristics and the molecular mechanism in the recognition of Tn-glycosylated MUC1 fragments by SN-101 differ entirely from those by SM3 and AR20.5. Intriguingly, it was reported that 237mAb, an antibody binds dominantly the Tn antigen of the glycopeptidic neoepitope found in the mouse tumour-associated glycoprotein podoplanin,^45^ does not recognise a conformational epitope. The crystal structure of 237mAb in complex with podoplanin glycopeptide indicates that the specific contacts between the antibody and the peptide moiety observed in a shallow groove can form only after the GalNAc enters the binding cavity.^46^ Notably, NMR studies showed that glycosylation does not provide any conformational effects in solution on the proximal peptide region of podoplanin. Furthermore, the podoplanin-derived glycopeptide bound to 237mAb has little regular secondary structure, demonstrating that the molecular mechanism in the interaction of 237mAb with the podoplanin glycopeptidic epitope differs completely from that of SN-101 with Tn-glycosylated MUC1 fragments.

## Conclusions

Given that many carbohydrate antigens such as Tn, T, ST, Lewis, and sialyl Lewis antigens are found not only in tumour cells but in embryonic and normal adult tissues,^47^ the advantages of targeting glycopeptidic neoantigens for cancer immunotherapy are evident.^48,49^ From the crystal structures of SN-101 and its complex with a glycopeptidic neoepitope and the results of epitope mapping analysis, we can now understand fully how SN-101 recognises cancer-relevant Tn-glycosylated MUC1 fragments. The structure of liganded SN-101 Fab provides a provocative template for engineering new antibodies exhibiting higher affinity and specificity to improve the clinical performance of anti-MUC1 mAbs. We believe that the present approach will accelerate dramatically the development of highly potential anti-cancer antibodies targeting neoantigens generated dynamically by aberrant and immature *O*-glycosylation in various cancer-relevant mucins and mucin-like domains widely distributed in the intrinsically disordered regions.^49,50^

## Experimental

### Synthesis of MUC1 glycopeptides

Microwave-assisted solid-phase syntheses of MUC1 glycopeptides/peptide derivatives **1–25** were performed with an EYELA microwave synthesizer Wave Magic MWS-1000A (Tokyo Rikakikai Co., LTD., Japan) according to the general methods reported previously.^11,20–23^ The characterisation data of compounds **1–25** are described in ESI Fig. S2–S26.†

### Generation of anti-MUC1 mAbs recognising glycopeptidic epitopes

MUC1 glycopeptide antigen **1** was conjugated with keyhole limpet hemocyanin (KLH) or aminooxy-functionalized nanoparticles^25–27^ by using the ketone linker and administered at the tail base of BDF-1 mice according to a protocol reported previously.^51^ Additional immunization was performed 17 days later using the same method. After 3 days, cells collected from iliac lymph nodes were fused with myeloma SP2 cells. The hybridomas were cultured in a HAT selective medium, and the antibody-producing cells were selected. Next, the hybridoma culture supernatant was subjected to assay using an ELISA plate displaying compounds **1** and **2** to test the ability to discriminate MUC1 glycopeptide epitope **1** from naked peptide **2**. SN-101 is one of the selected clones to produce antibodies exhibiting the ability needed for a criterion of the first screening (Fig. 1). Large-scale preparation of SN-101 was performed by using the standard ascites antibody production method.^52,53^ Briefly, hybridoma cells (0.5 mL) were injected into a BALB/c nu–nu mouse and the mixture containing the antibody was precipitated from collected ascites by the addition of a 2/3 volume of saturated ammonium sulphate solution at 4 °C. The precipitates were dissolved in water and dialyzed against a buffer (0.75 M glycine, 1.5 M NaCl, and pH 8.9). The crude mixture was applied to a protein A column (Protein A-Sepharose Fast Flow, GE Healthcare), and the SN-101 antibody was eluted with a buffer (0.1 M glycine-HCl, 0.1 M NaCl buffer, and pH 3.0) and the fraction containing the antibody was neutralised by using 2 M Tris–HCl buffer (pH 9.0).

### Preparation and crystallization of SN-101 Fab

SN-101 (IgG1) (10 mL, 4 mg mL^−1^) was dialyzed for 24 h into a solution of 5 mM EDTA, 25 mM cysteine and 10 mM citrate (pH 6.5). The solution was added to immobilised ficin (Thermo Scientific) using a ratio of 24 mg of IgG/1 mL of ficin resin^54^ and was incubated for 5 h with an end-over-end mixer at 37 °C. The reaction mixture was filtered to remove the resin and dialysed into 20 mM sodium phosphate buffer (pH 7.0). Affinity chromatography was performed using a Hi trap Protein G column (1 mL) to remove the Fc fragments from the reaction mixture. The Fab and undigested IgG1 were eluted with acidic buffer (0.1 M glycine-HCl, pH 2.7) as a single peak from the column. Subsequently, SN-101 Fab was isolated by size exclusion chromatography using a HiLoad 16/600 Superdex 75 column (GE Healthcare) under 50 mM Tris–HCl buffer (pH 8.0) containing 200 mM NaCl. SN-101 Fab was concentrated to 15 mg mL^−1^ and used directly for the crystallisation to obtain the crystals of unliganded SN-101 Fab. In addition, SN-101 Fab was mixed with MUC1 glycopeptide **3** in a 1 : 5 molar ratio for SN-101/MUC1 glycopeptide **3** (SN-101–MUC1). The crystallisation screening was performed using the sitting-drop vapor-diffusion method with a series of crystallisation kits from Qiagen (Hilden, Germany). Protein solution (0.2 μL) was mixed with an equal volume of reservoir solution at 20 °C. Appropriate crystals of the unliganded form and MUC1-liganded SN-101 Fab were obtained in 300–350 mM NaSCN and 20–30% (w/w) PEG3350.

### Data collection, structural determination, and refinement

Crystals were soaked in cryo-protectant solution (20% glycerol in reservoir solution), followed by flash-cooling under a stream of liquid nitrogen at −183 °C. Diffraction data were collected on beamline BL17A at the Photon Factory (Tsukuba, Japan). All data were processed and scaled using XDS.^55^ All data collection statistics are summarized in ESI Table S1.† The structures of the unliganded form and SN-101–MUC1 complex were determined by the molecular replacement method with the program PHASER.^56^ The structure of the unliganded form was solved using the structure of antibody SYA/J6 (PDB code: 1PZ5) as a search model. Subsequently, the structure of the SN-101–MUC1 complex was determined using the structure of the unliganded form as a search model. Several rounds of refinement were performed using the program Phenix refine^57^ in the Phenix program suite, alternating with manual fitting and rebuilding based on 2*F*_o_ − *F*_c_ and *F*_o_ − *F*_c_ electron density using COOT.^58^ Then, water molecules and MUC1 glycopeptide **3** were built based on 2*F*_o_ − *F*_c_ and *F*_o_ − *F*_c_ electron densities. The final refinement statistics and geometry defined by using MolProbity^59^ are shown in ESI Table S1.† All structural figures were generated by using PyMol (W. L. DeLano, The PyMOL Molecular Graphics System, Version 1.7.4 Schrödinger, LLC, 2002).

### Binding assay using glycopeptide microarray

According to a procedure reported previously,^11,42,43^ AO/PC-coated plastic slides were activated by 2 N HCl treatment for 4 h at 37 °C. Next, MUC1 glycopeptides/peptide derivatives **4–24** were spotted in quadruplicate at three different concentrations (1, 10, and 100 μM) in 25 mM acetate buffer, pH 5.0. The spotted slides were incubated for 1 h at 80 °C to complete the immobilisation and then dropped into an aqueous solution of succinic anhydride (10 mg mL^−1^) for 1 h at room temperature to cap unreacted aminooxy groups. A silicone rubber sheet with chambers was attached to the slide surface. Then, a cover glass was set on each well. Subsequently, SN-101 solution (20 μg mL^−1^) in reaction buffer was added through the interstice. After 2 h incubation at room temperature in a humidified chamber, a Cy3-labeled goat anti-mouse IgG antibody (20 μg mL^−1^) was added to the interstice, and the slide was incubated for 1 h at room temperature. After incubation, each well was washed twice. Fluorescence images of the microarray slides were obtained by using a GlycoStation™ Reader 1200 (GlycoTechnica Ltd., Japan). The digital images of fluorescence responses were analysed using ArrayVision™ software version 8.0 (GE Healthcare) and GraphPad Prism™ software version 5 (GrapfPad Software, Inc.). The net intensity value was analysed with Array Vison software, subtracting the background value. The average relative fluorescence unit (RFU) was plotted as a histogram. The error bars represent the standard deviation.

### Binding analysis using SPR

The binding affinity of SN-101 was measured with a surface plasmon resonance instrument (Biacore 2000, GE Healthcare). First, MUC1 glycopeptide **25** was immobilised on a CM5-sensor chip using a standard maleimide coupling method. The resonance units (RUs) of the sensor chip increased to 31 RU after immobilisation. In addition, a cell that was blocked with l-cystein was prepared as a reference cell. SN-101 antibody Fab was dissolved in HBS-EP buffer and then 5.0 μM, 2.5 μM, 1.3 μM, 0.63 μM, 0.31 μM, 0.16 μM, 0.08 μM, and 0.04 μM of Fab solutions were prepared. A Fab solution was injected at a flow rate of 20 μL min^−1^. The data were analysed with BIA evaluation version 4.1 software (GE Healthcare). The affinity constant (*K*_D_) of SN-101 Fab with MUC1 glycopeptide **25** was calculated from kinetic parameters (association and dissociation).

### Interaction of SN-101 with cancer cell surface MUC1

Human breast cancer cells, OCUB-M established from the pleural effusion of a 53 year old Japanese female with recurrent breast cancer (RRID: CVCL_1621),^33^ were cultured at 37 °C for 24 h in D-MEM in the presence of 10% fetal bovine serum (FBS) under a 5% CO_2_ atmosphere. The cells were washed with 0.01 M PBS and treated with 0.01 M PBS containing 4% paraformaldehyde. After 10 min, the culture solution was replaced with 0.01 M PBS containing 0.1% Triton X100, and then the cells were treated for 20 min with blocking buffer (0.01 M PBS containing BSA). Next, the cells were kept in the blocking buffer in the presence or absence of SN-101 (10 μg mL^−1^) for 1 h. After washing with 0.01 M PBS, the cells were treated with a solution containing an Alexa FluorR555 labelled anti-mouse IgG antibody (2 μg mL^−1^). After washing with 0.01 M PBS, the cancer cells were subjected to observation by using a BZ-9000 all-in-one fluorescence microscope (KEYENCE). The inhibitory effect of SN-101 on the proliferation of the OCUB-M cells was assessed preliminarily by using Cell Titer 96 Aqueous One Solution Cell Proliferation Assay (Promega). The cultured breast cancer OCUB-M cells (3 × 10^3^ cells) were co-incubated at 37 °C for 48 h with SN-101 (0.565 mg mL^−1^ and 1.13 mg mL^−1^) in D-MEM containing 10% FBS under a 5% CO_2_ atmosphere. Then, the cells were added with the above assay reagent (20 μL) per well and further cultured at 37 °C for 2 h under a 5% CO_2_ atmosphere. Finally, cell viability was determined by measuring the absorbance at 490 nm in comparison with control wells. The results demonstrated that SN-101 (1.13 mg mL^−1^) exhibits 95% growth inhibition when compared with controls, while this inhibitory effect is reduced to 45% in the presence of SN-101 (0.565 mg mL^−1^).

## Conflicts of interest

There are no conflicts to declare.

## Supplementary Material

SC-011-D0SC00317D-s001
