# Correction: A straightforward approach to antibodies recognising cancer specific glycopeptidic neoepitopes

**DOI:** 10.1039/d0sc90254c

**Published:** 2020-11-18

**Authors:** Hajime Wakui, Yoshikazu Tanaka, Toyoyuki Ose, Isamu Matsumoto, Koji Kato, Yao Min, Taro Tachibana, Masaharu Sato, Kentaro Naruchi, Fayna Garcia Martin, Hiroshi Hinou, Shin-Ichiro Nishimura

**Affiliations:** Field of Drug Discovery Research, Faculty of Advanced Life Science, Graduate School of Life Science, Hokkaido University N21 W11, Kita-ku Sapporo 001-0021 Japan shin@sci.hokudai.ac.jp; Graduate School of Life Sciences, Tohoku University 2-1-1 Katahira, Aoba-ku Sendai 980-8577 Japan; Field of X-ray Structural Biology, Faculty of Advanced Life Science, Graduate School of Life Science, Hokkaido University N10 W8, Kita-ku Sapporo 060-0810 Japan; Research Institute for Interdisciplinary Science and Graduate School of Natural Science and Technology, Okayama University 3-1-1, Tsushima-naka, Kita-ku Okayama 700-8530 Japan; Department of Bioengineering, Graduate School of Engineering, Osaka City University Sumiyoshi-ku Osaka 558-8585 Japan; Medicinal Chemistry Pharmaceuticals, Co., Ltd. N9 W15, Chuo-ku Sapporo 060-0009 Japan

## Abstract

Correction for ‘A straightforward approach to antibodies recognising cancer specific glycopeptidic neoepitopes’ by Hajime Wakui *et al.*, *Chem. Sci.*, 2020, **11**, 4999–5006, DOI: 10.1039/D0SC00317D.

The authors regret that part Fig. 1b was missing from the version of Fig. 1 shown in the original article. The correct version of Fig. 1 is presented below.

**Fig. 1:**
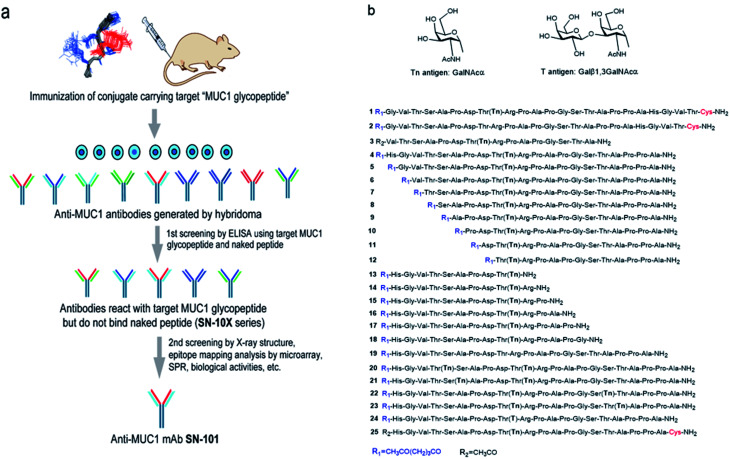
Generation of epitope-defined anti-MUC1 antibodies. (a) A strategy for the generation of antibodies targeting glycopeptidic epitopes by using synthetic glycopeptides designed for the streamlined process from the immunization of “conformational glycopeptidic neoepitopes”, antibody selection, and characterization. (b) A list of compounds used in this study. Compound **1** was conjugated with KLH by using the Cys residue (red) or aminooxy-functionalized nanoparticles^25–27^ by using the ketone linker (blue) and used for the immunization. The first screening was performed by ELISA immobilizing compounds **1** and **2** using Cys residue (red) to collect antibodies binding selectively with glycopeptide **1**. Compound **3** was used for the co-crystallization with SN-101. Compounds **4–24** were displayed on the microarray by means of the ketone linker (blue) and employed for epitope mapping analysis. Compound **25** was used for the SPR analysis by immobilizing with Cys residue (red).

The Royal Society of Chemistry apologises for these errors and any consequent inconvenience to authors and readers.

