# Supplementary material for: A straightforward approach to antibodies recognising cancer specific glycopeptidic neoepitopes
Source: Chem Sci. 2020 Apr 30;11(19):4999–5006. doi: 10.1039/d0sc00317d (PMC8159228; doi:10.1039/d0sc00317d)
Supplement: SC-011-D0SC00317D-s001 [file SC-011-D0SC00317D-s001.pdf]

Electronic Supplementary Material (ESI) for Chemical Science.
This journal is © The Royal Society of Chemistry 2020

# A straightforward approach to antibodies recognising cancer specific glycopeptidic neoepitopes


| **Supplementary data** | | Page(s) |
|---|---|---|
| Supplementary Methods | | S2 |
| Supplementary Results | | S3~S27 |
| Table S1 | Data collection and refinement statistics | S3~S4 |
| Figure S1 | Sequence alignment of anti-MUC1 mAbs | S5 |
| Figure S2~26 | Characterization of compounds **1~25** | S1~30 |
| Supplementary References | | S36~37 |

## Supplementary Methods

### General methods and materials

All solvents and reagents were purchased from Aldrich Chemical Co. (Milwaukee, WI), Tokyo Chemical Industry Co. Inc. (Tokyo, Japan), Wako Pure Chemical Industries Co. Inc. (Tokyo, Japan), Funakoshi Co., Inc. (Tokyo, Japan), Medicinal Chemistry Pharmaceuticals, Co., Inc. (Sapporo, Japan), Thermo Fisher Scientific K. K. Co. Inc. (Tokyo, Japan), Kanto Chemical Co. Inc. (Tokyo, Japan) and used without further purification. Fmoc-based solid phase peptide synthesis was performed with NovaPEG Rink Amide resin (loading 0.45 mmol/g). Fmoc-L-amino acids, except for glycosylated compounds, were purchased from Novabiochem (Darmstadt, Germany) and Watanabe chemical industries (Japan). Cy3$^{TM}$-labeled goat anti-mouse IgG antibody was purchased from (Jackson ImmunoResearch). Microarray slides (75×25×1 mm) were supplied from Sumitomo Bakelite Co., Ltd. (Tokyo, Japan). Fmoc-($Ac_3$GalNAcα1→)serine/threonine, Fmoc-($Ac_4$Galβ1,3$Ac_2$GalNAcα1→)serine/threonine were synthesized according to previously reported methods [S1-S3]. Preparative HPLC purifications were performed using Hitachi system equipped with L-7100 pump and L-7405 UV detector using a reverse-phase C18 column Inertsil ODS-3 250×4.6 mm I.D. (GL Sciences Inc., Tokyo, Japan). Analytical HPLC were performed using a Prominence Shimadzu HPLC system [(Shimadzu Corporation, Kyoto, Japan) with LC-20B pump, SPD-20A UV/VIS detector at 220 nm for monitor and Inertsil ODS-3 reversed-phase C-18 column C18 ODS-3 250×20 mm I.D (GL Sciences Inc., Tokyo, Japan). Matrix Assisted Laser Desorption Ionization time-of-flight mass (MALDI-TOFMS) spectra were obtained with an Ultraflex III TOF mass spectrometer (Bruker Daltonik GmbsH Co. Inc, Germany) equipped with a reflector and controlled by the Flexcontrol 3.0 software package.

## Supplementary Results

**Table S1**. Data collection and refinement statistics

| | **SN-101-MUC1 complex** | **SN-101 non-liganded** |
|---|---|---|
| **Data collection** | | |
| Beamline | Photon Factory BL-17A | Photon Factory BL-17A |
| Resolution range | 50 - 1.77 (1.88 - 1.77) | 50 - 2.40 (2.55 - 2.40) |
| Space group | *C* 1 2 1 | *C* 1 2 1 |
| Unit cell | *a* = 159.92, *b* = 39.98, *c* = 63.87, β = 100.56 | *a* = 159.86, *b* = 40.1, *c* = 67.78, β = 99.83 |
| Unique reflections | 38810 (6165) | 16818 (2665) |
| Completeness (%) | 99.6 (98.5) | 99.6 (98.9) |
| $I/\sigma(I)$ | 15.3(1.97) | 12.78(2.36) |
| Wilson *B*-factor | 26.42 | 42.14 |
| $R_{meas}$ (%) | 7.1 (92.6) | 11.3 (74.2) |
| Redundancy | 4.98 (4.95) | 4.99 (5.08) |
| *CC*1/2 (%) | 99.9 (72.3) | 99.7 (76.1) |
| **Refinement** | | |
| Resolution range (Å) | 39.3 - 1.77 (1.84 – 1.77) | 38.9 - 2.40 (2.49 – 2.40) |
| Reflections used in refinement | 38800 (3774) | 16813 (1659) |
| $R_{work}$ | 0.1770 (0.3304) | 0.2253 (0.2642) |
| $R_{free}$ | 0.2286 (0.3461) | 0.2778 (0.3206) |
| Number of atoms | 3619 | 3348 |
| macromolecules | 3358 | 3295 |

| | | |
|---|---|---|
| ligands | 24 | 16 |
| solvent | 237 | 37 |
| *B*-factors (Å$^2$) | 31.94 | 51.59 |
| Macromolecules (Å$^2$) | 31.54 | 51.58 |
| Ligands (Å$^2$) | 39.20 | 70.60 |
| Solvent (Å$^2$) | 36.91 | 43.72 |
| **r.m.s.d (Å)** | | |
| RMS (bonds) | 0.011 | 0.003 |
| RMS (angles) | 1.12 | 0.63 |
| **Ramachandran plot (%)** | | |
| Ramachandran favoured | 97.44 | 96.22 |
| Ramachandran allowed | 2.56 | 3.78 |
| Ramachandran outliers | 0.00 | 0.00 |

**Figure S1**. Sequence alignment of anti-MUC1 mAbs SM3, SN-101, and AR20.5. Complementarity Determining Region (CDR) is colored with light brown. Residues with green background interact with MUC1 peptide sequences *via* hydrogen bonds. Residues with orange background interact with glycan moiety (GalNAc).

```
H chain
        1        10        20        30        40        50        60
SM3     VQLQESGGGLVQPGGSMKLSCVASGFTFSNYWMNWVRQSPEKGLEWVAEIRLKSNNYATH
SN-101  VNLEESGGGLVQPGGSMKLSCVASGFTFSNYWMNWVRQSPEKGLEWVAQIRLKSDNYATH
AR20.5  VKLVESGGGLVAPGGSLKLSCAASGFTFSSYPMSWVRQTPEKRLEWVAYINNGGG--NPY
          * ******* **** **** ******* * * **** *** ***** *

        61       70        80        90        100       110       120
SM3     YAESVKGRFTISRDDSKSSVYLQMNNLRAEDTGIYYCTG-VGQFAYWGQGTTVTVSIVVT
SN-101  YAESVKGRFTISRDDSKSSVYLQMNNLRAEDTGIYYCTGGV--FDYWGQGTTLTVSSSKT
AR20.5  YPDTVKGRFTISRDNAKNTLYLQMSSLKSEDTAIYYCIRQYYGFDYWGQGTTLTVSSAKT
        *   **********  *   ****  *  *** ****      * ******* ***   *

L chain
        1        10        20        30        40        50        60
SM3     ----------LTTSPGETVTLTCRSSTGAVT--TSNYANWVQEKPDHLFTGLIGGTNNRA
SN-101  DVVMTQTPLSLPVSLGDQASISCRSSQSLVHSNGNTYLHWYLQKPGQSPKLLIYKVSNRF
AR20.5  DVLMTQTPLSLPVSLGDQASISCRSSQTIVHSNGKIYLEWYLQKPGQSPKLLIYRVSKRF
                  *  * *      ****   *      *  *   **      **     *

        61       70        80        90        100       110
SM3     PGVPARFSGSLIGDKAALTITGAQTEDEAIYFCALWY-SNHWVFGGGTKLTV
SN-101  SGVPDRFSGSGSGTDFTLKISRVEAEDLGVYFCSQSTHVPPWTFGGGTKLEI
AR20.5  SGVPDRFSGSGSGTDFTLKISRVEAEDLGVYYCFQGSHV-PWTFGGGTKLEI
         *** *****  *    * *     **   * *        *.*******
```

**Figure S2**. Characterization of compound **1**. Analytical HPLC: A : B ($H_2O$ in 0.1%TFA : $CH_3CN$ in 0.1%TFA) A/B gradient 0~50 min, 10~35% B. MALDI-TOFMS: $C_{108}H_{179}N_{31}O_{39}S$, theoretical mass: 2560.223, observed mass: 2561.275 [M+H]$^+$

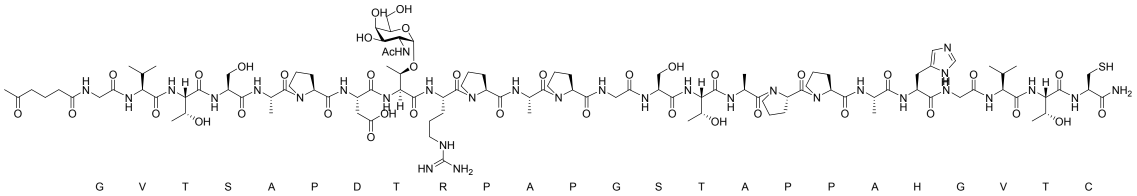


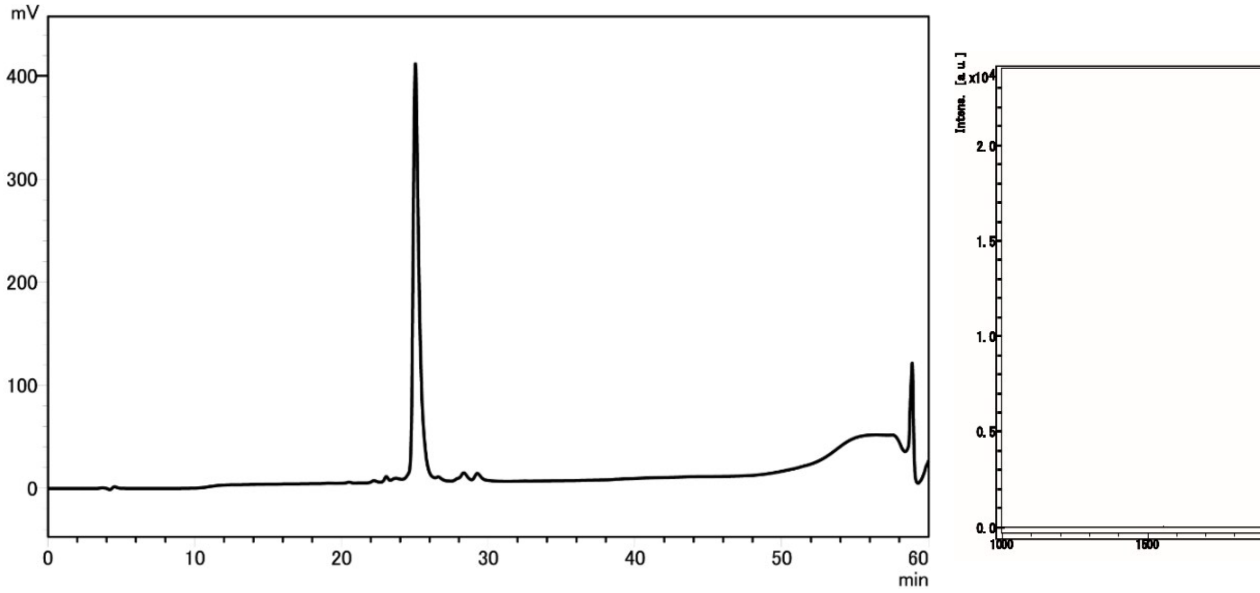


**Figure S3**. Characterization of compound **2**. Analytical HPLC: A : B ($H_2O$ in 0.1%TFA : $CH_3CN$ in 0.1%TFA) A/B gradient 0~50 min, 10~35% B. MALDI-TOFMS: $C_{100}H_{160}N_{30}O_{34}S$, theoretical mass: 2357.143, observed mass: 2358.627 [M+H]$^+$

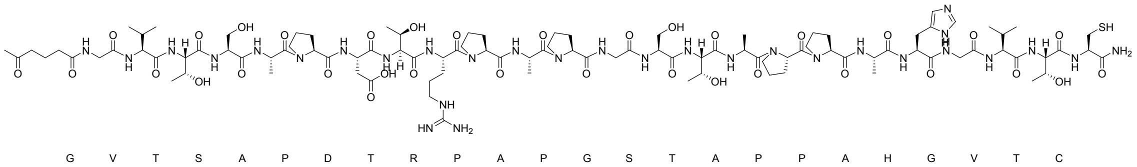

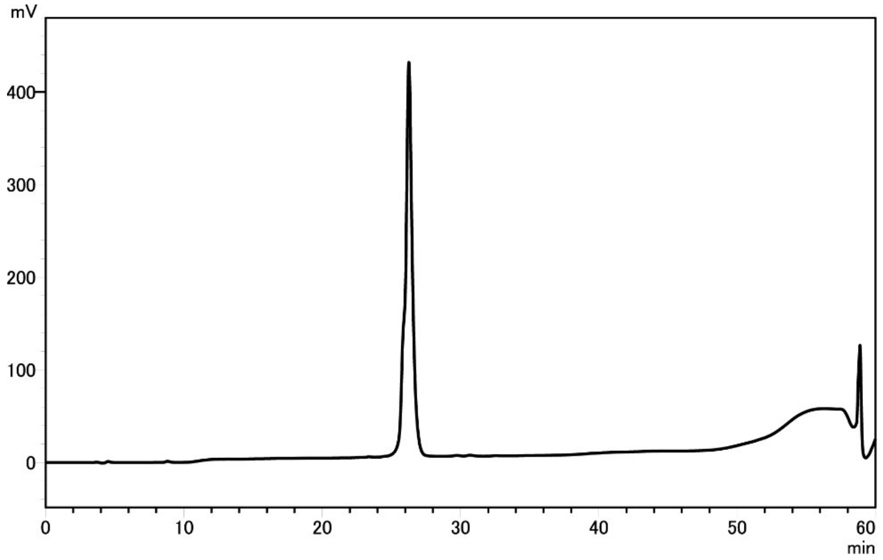


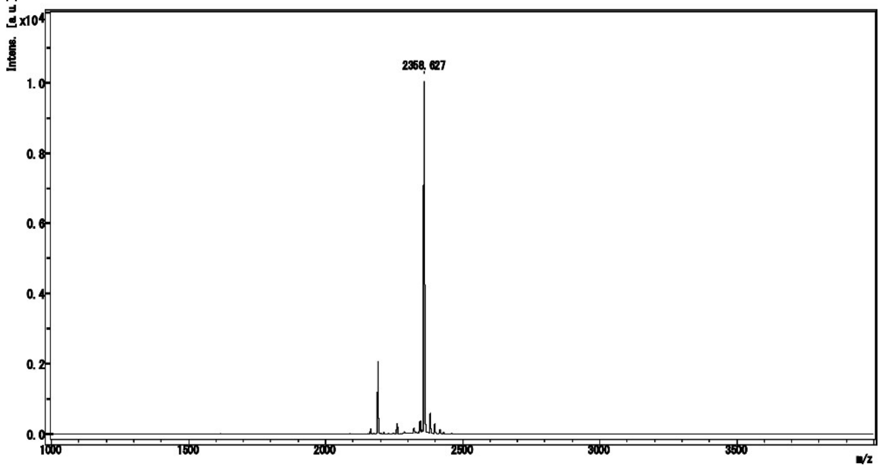


**Figure S4**. Characterization of compound **3**. Analytical HPLC: A : B ($H_2O$ in 0.1%TFA : $CH_3CN$ in 0.1%TFA) A/B gradient 0~50 min, 10~35% B. MALDI-TOFMS: $C_{94}H_{149}N_{27}O_{34}$, theoretical mass: 2200.076, observed mass: 2202.146 $[M+H]^+$

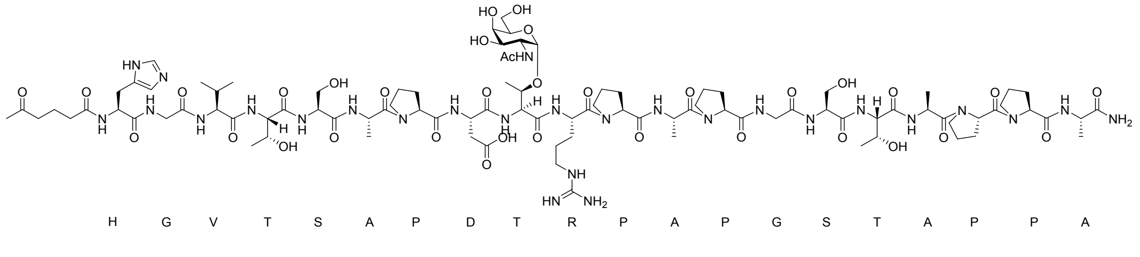

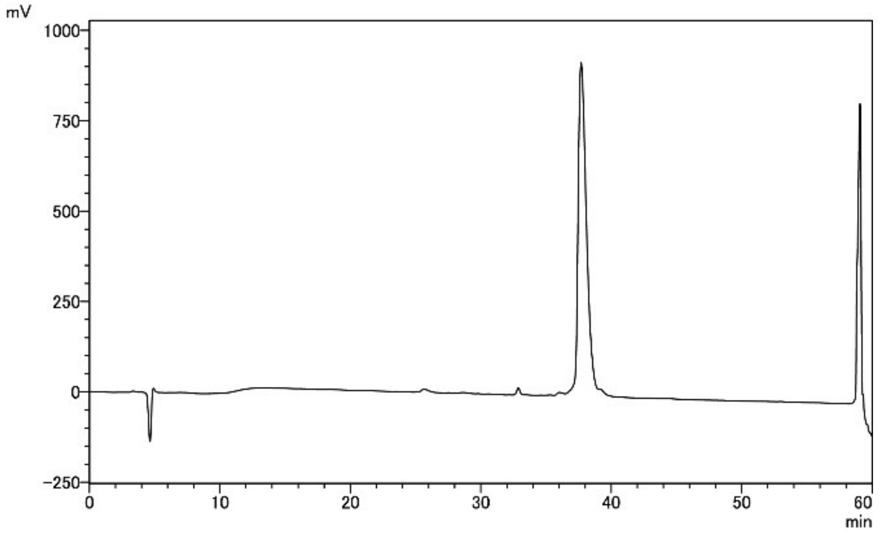
mV
1000
750
500
250
0
−250
0
10
20
30
40
50
60
min

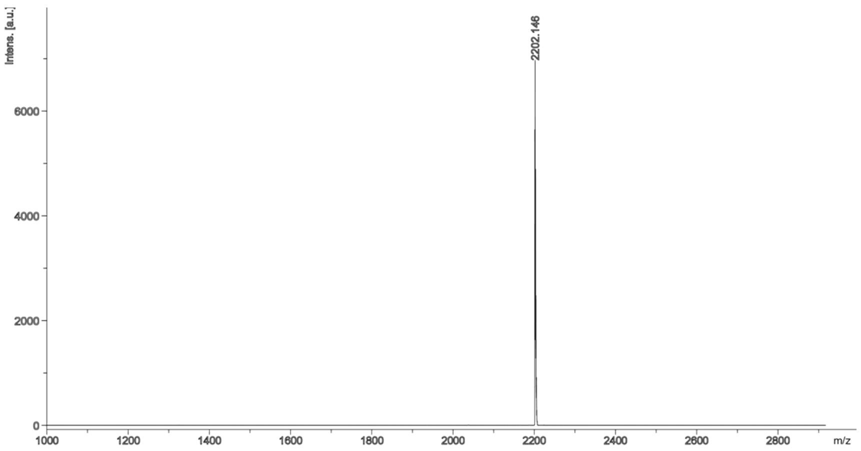
Intens. [a.u.]
2202.146
6000
4000
2000
0
1000
1200
1400
1600
1800
2000
2200
2400
2600
2800
m/z

**Figure S5**. Characterization of compound **4**. Analytical HPLC: A : B ($H_2O$ in 0.1%TFA : $CH_3CN$ in 0.1%TFA) A/B gradient 0~50 min, 10~35% B. MALDI-TOFMS: $C_{88}H_{142}N_{24}O_{33}$, theoretical mass: 2063.017, observed mass: 2064.098 $[M+H]^+$

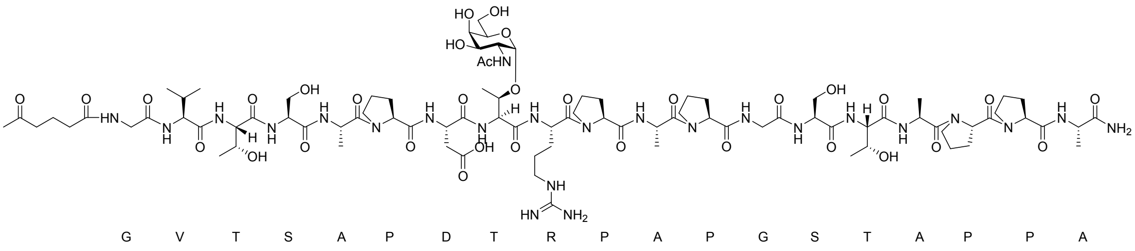


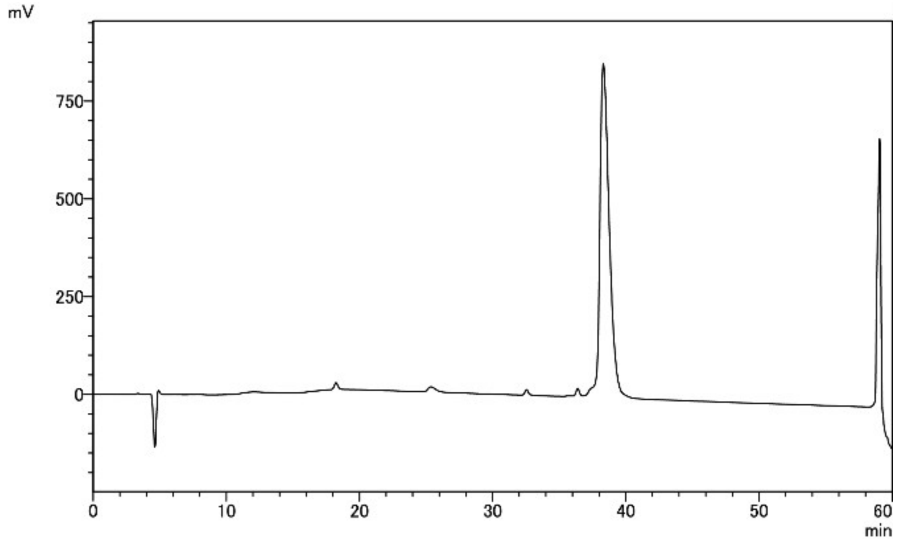


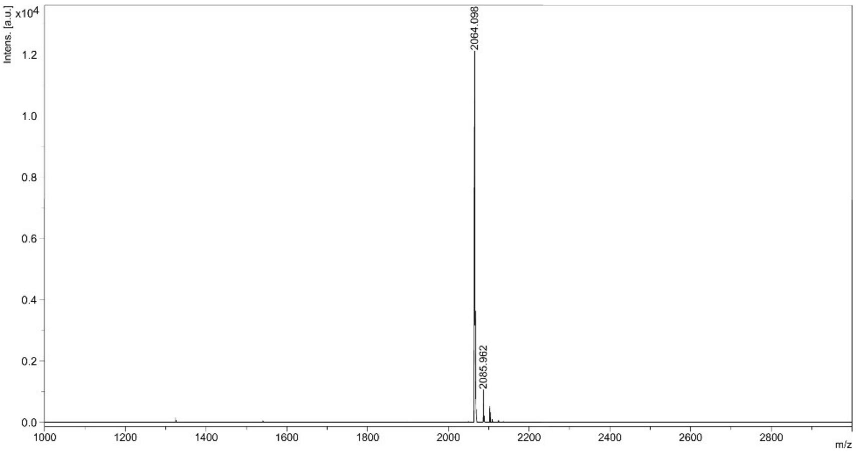

**Figure S6**. Characterization of compound **5**. Analytical HPLC: A : B ($H_2O$ in 0.1%TFA : $CH_3CN$ in 0.1%TFA) A/B gradient 0~50 min, 10~35% B. MALDI-TOFMS: $C_{86}H_{139}N_{23}O_{32}$, theoretical mass: 2005.996, observed mass: 2007.132 $[M+H]^+$

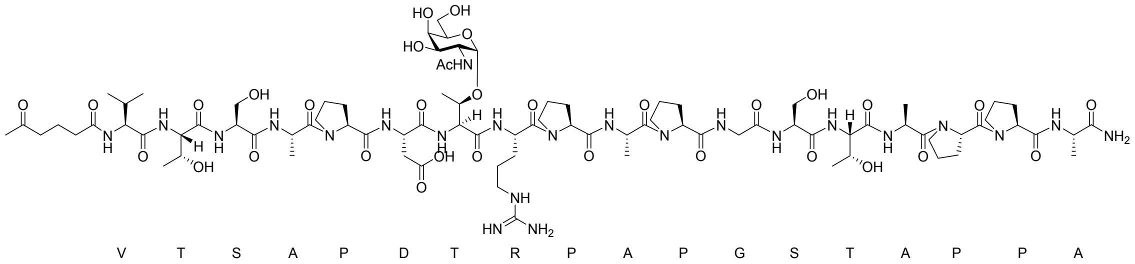


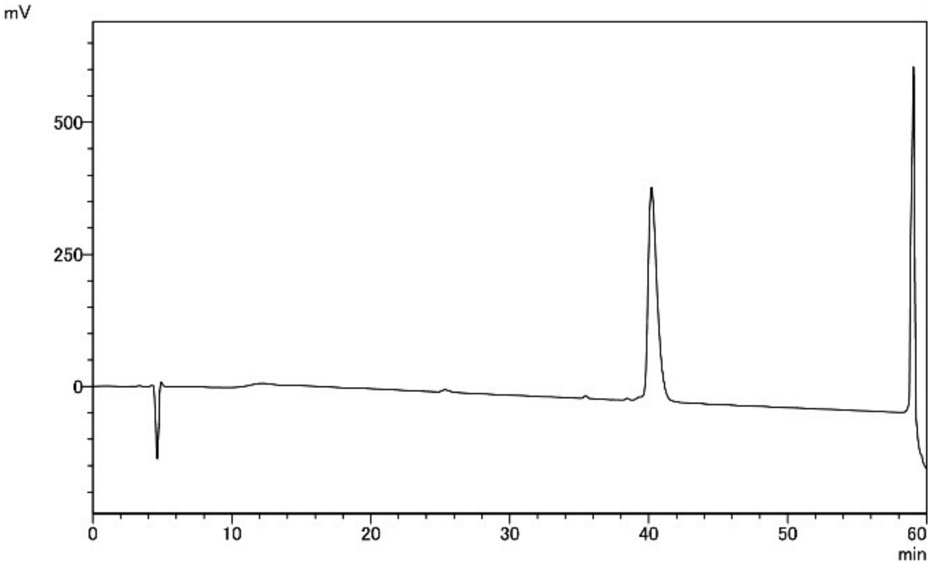


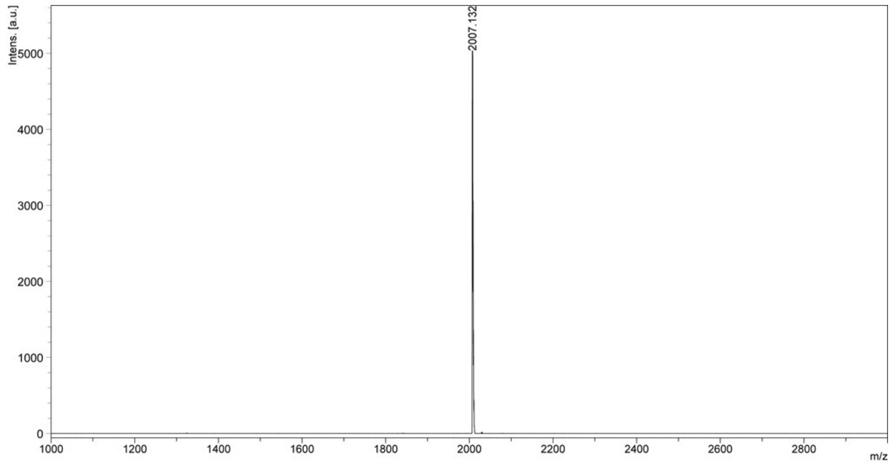

**Figure S7**. Characterization of compound **6**. Analytical HPLC: A : B ($H_2O$ in 0.1%TFA : $CH_3CN$ in 0.1%TFA) A/B gradient 0~50 min, 10~35% B. MALDI-TOFMS: $C_{81}H_{130}N_{22}O_{31}$, theoretical mass: 1906.927, observed mass: 1907.954 $[M+H]^+$

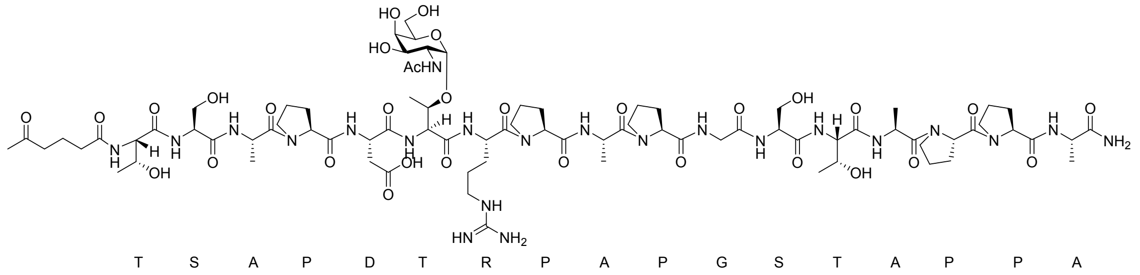


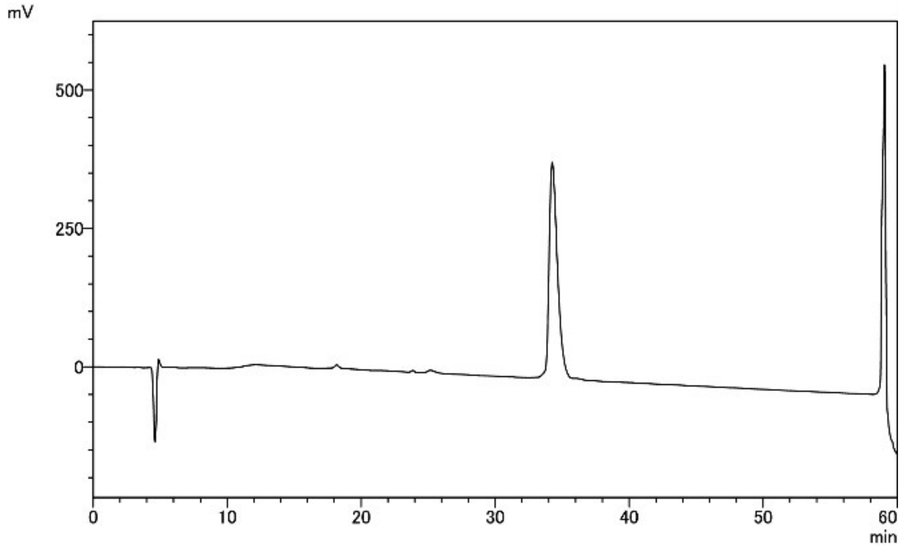


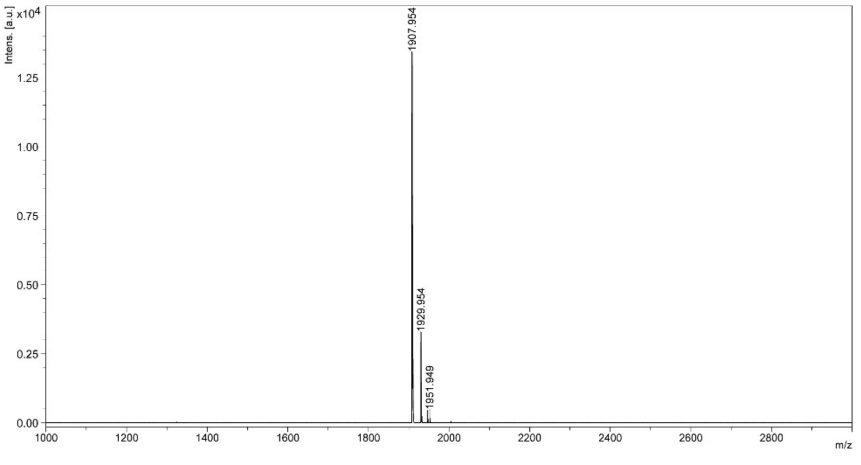

**Figure S8**. Characterization of compound **7**. Analytical HPLC: A : B ($H_2O$ in 0.1%TFA : $CH_3CN$ in 0.1%TFA) A/B gradient 0~50 min, 10~35% B. MALDI-TOFMS: $C_{77}H_{123}N_{21}O_{29}$, theoretical mass: 1805.880, observed mass: 1806.896 $[M+H]^+$

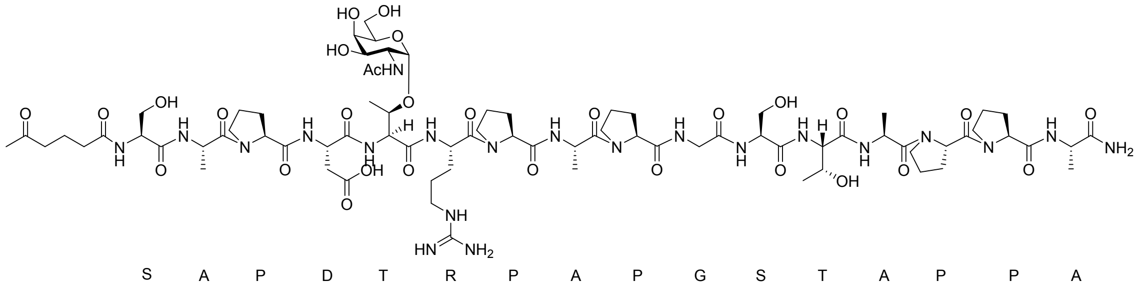


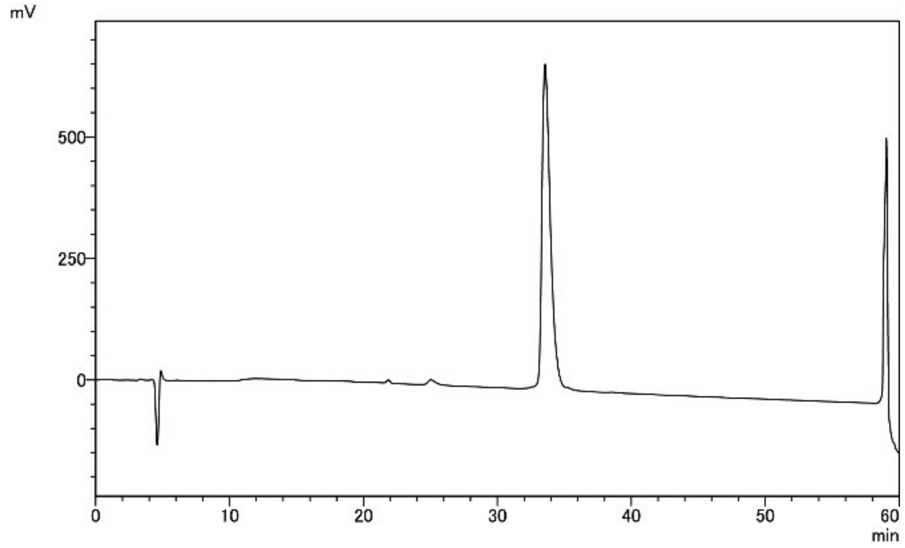


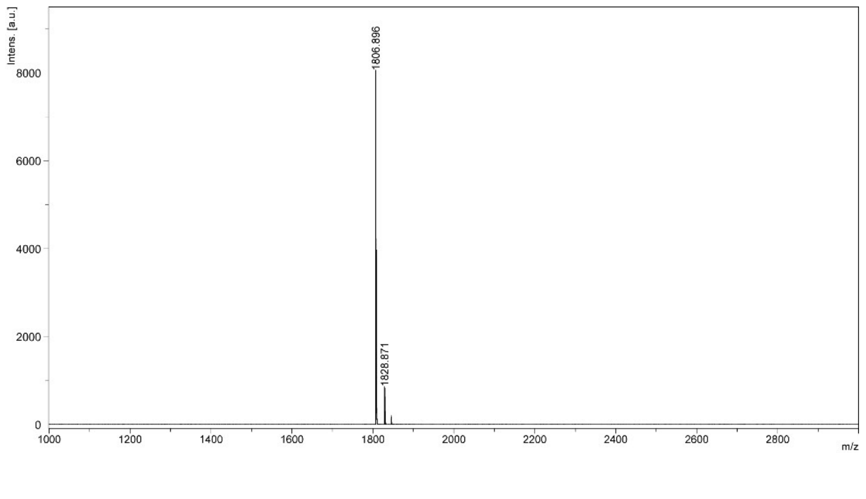

**Figure S9**. Characterization of compound **8**. Analytical HPLC: A : B ($H_2O$ in 0.1%TFA : $CH_3CN$ in 0.1%TFA) A/B gradient 0~50 min, 10~35% B. MALDI-TOFMS: $C_{74}H_{118}N_{20}O_{27}$, Theoretical mass: 1718.884, Observed mass: 1719.861 $[M+H]^+$

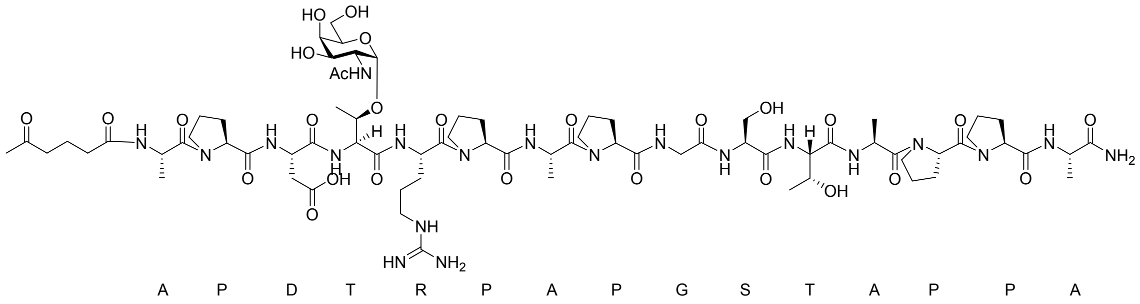


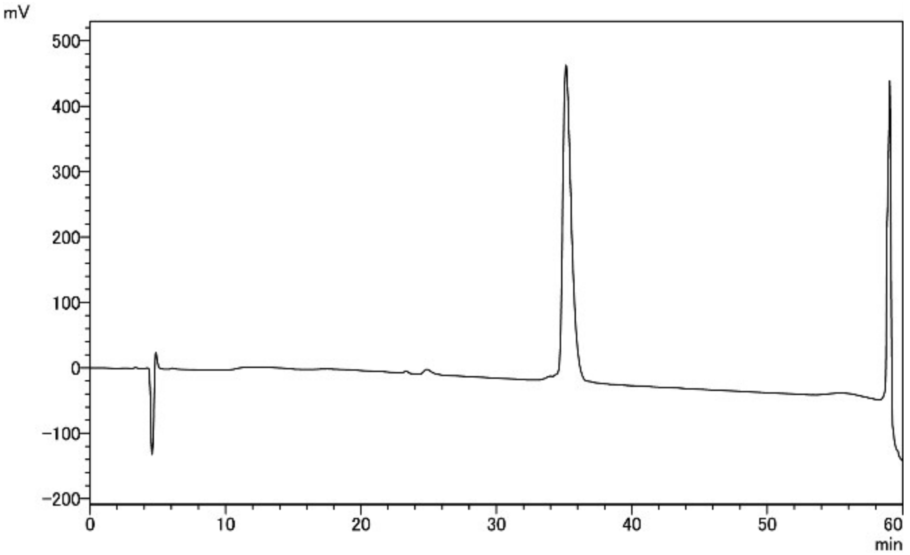


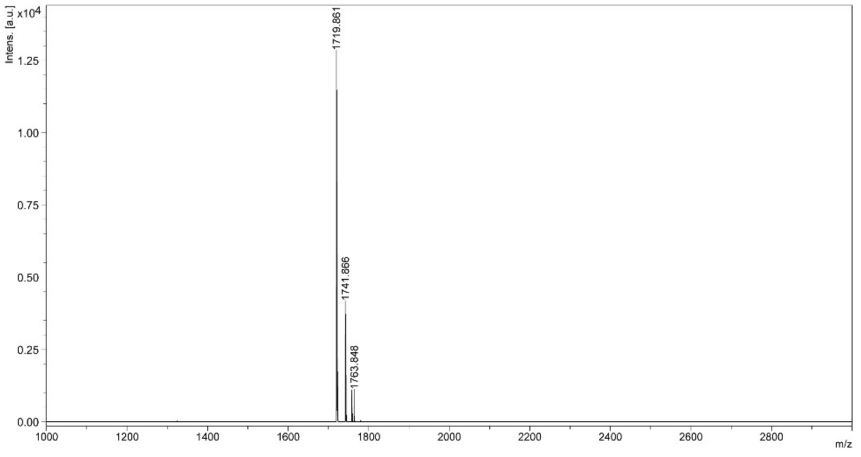

**Figure S10**. Characterization of compound **9**. Analytical HPLC: A : B ($H_2O$ in 0.1%TFA : $CH_3CN$ in 0.1%TFA) A/B gradient 0~50 min, 10~35% B. MALDI-TOFMS: $C_{71}H_{113}N_{19}O_{26}$, theoretical mass: 1647.810, observed mass: 1848.878 $[M+H]^+$

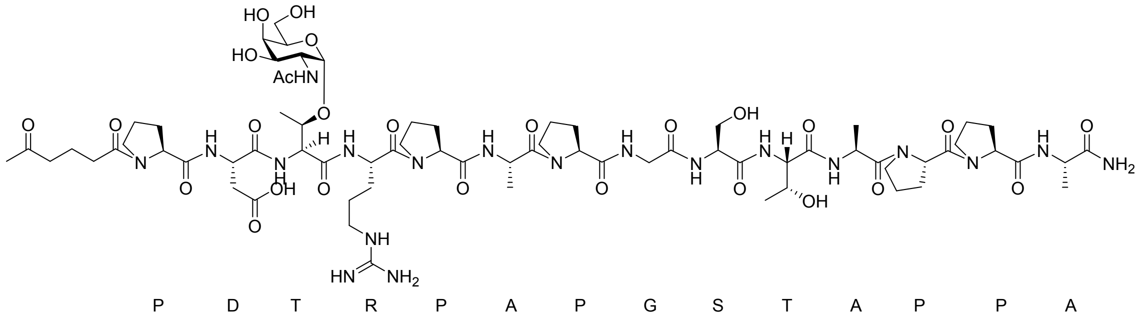


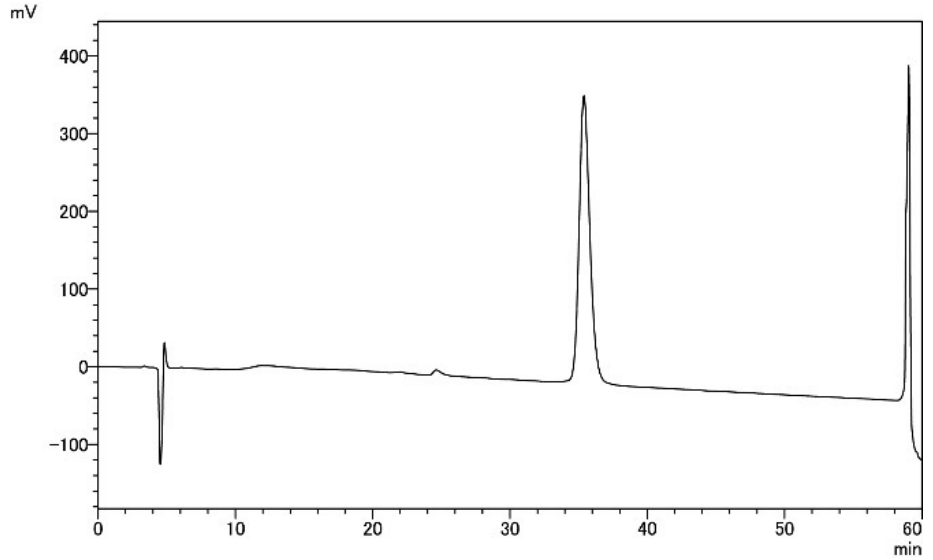


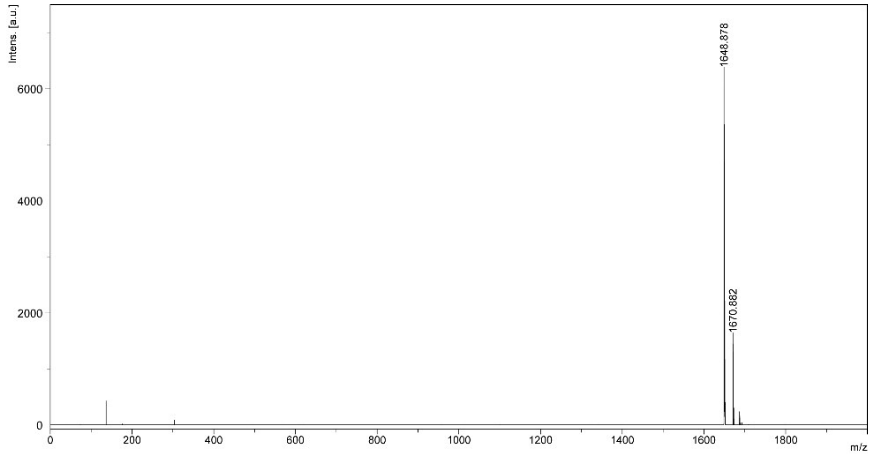

**Figure S11**. Characterization of compound **10**. Analytical HPLC: A : B ($H_2O$ in 0.1%TFA : $CH_3CN$ in 0.1%TFA) A/B gradient 0~50 min, 10~35% B. MALDI-TOFMS: $C_{66}H_{106}N_{18}O_{25}$, theoretical mass: 1550.758, observed mass: 1551.800 $[M+H]^+$

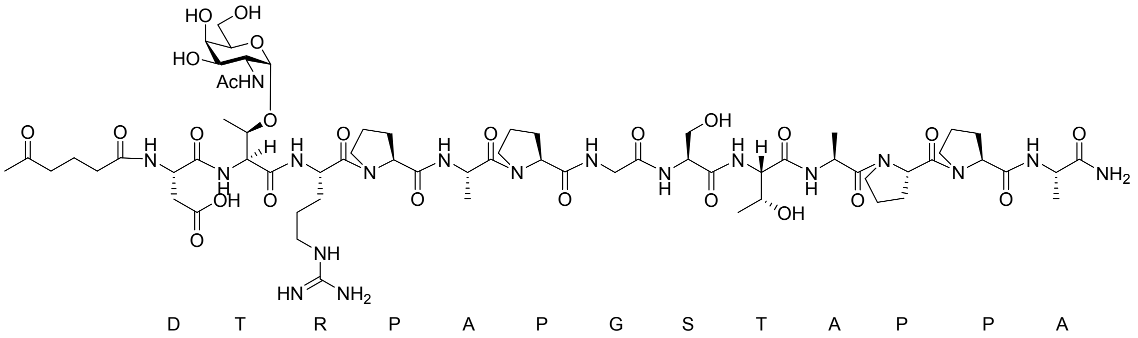


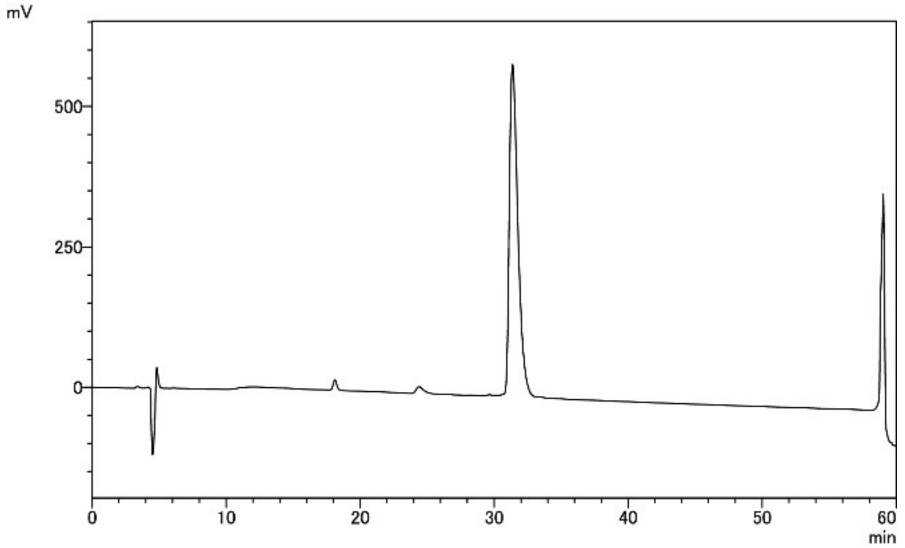


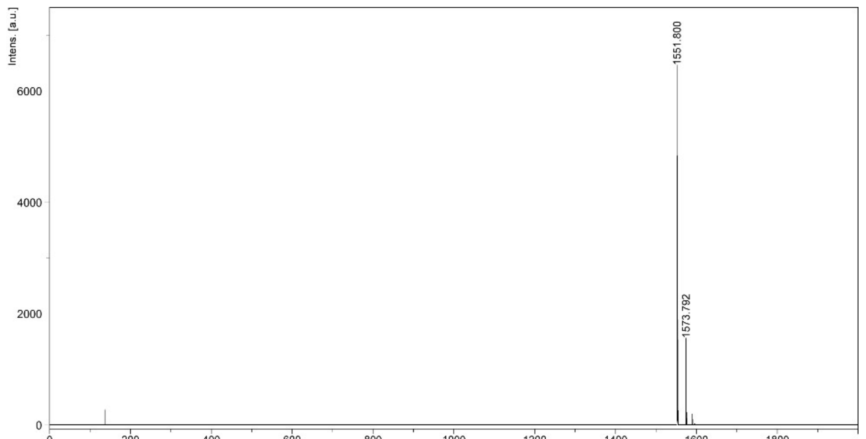

**Figure S12**. Characterization of compound **11**. Analytical HPLC: A : B ($H_2O$ in 0.1%TFA : $CH_3CN$ in 0.1%TFA) A/B gradient 0~50 min, 10~35% B. MALDI-TOFMS: $C_{62}H_{101}N_{17}O_{22}$, Theoretical mass: 1435.731, Observed mass: 1436.795 $[M+H]^+$

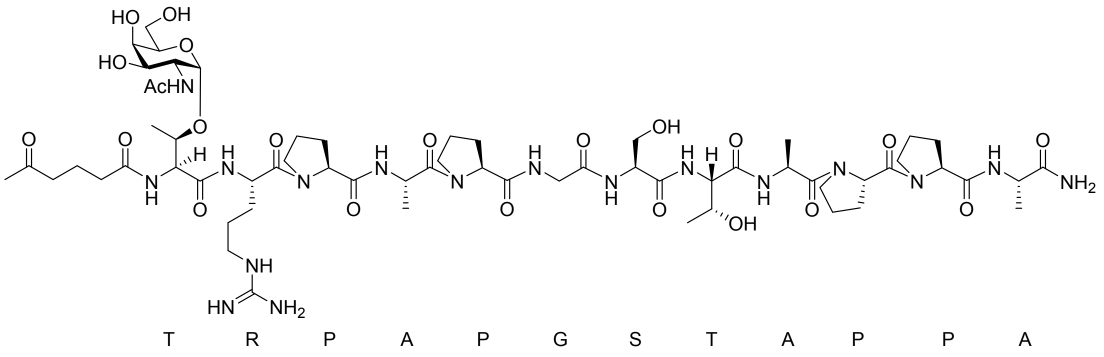


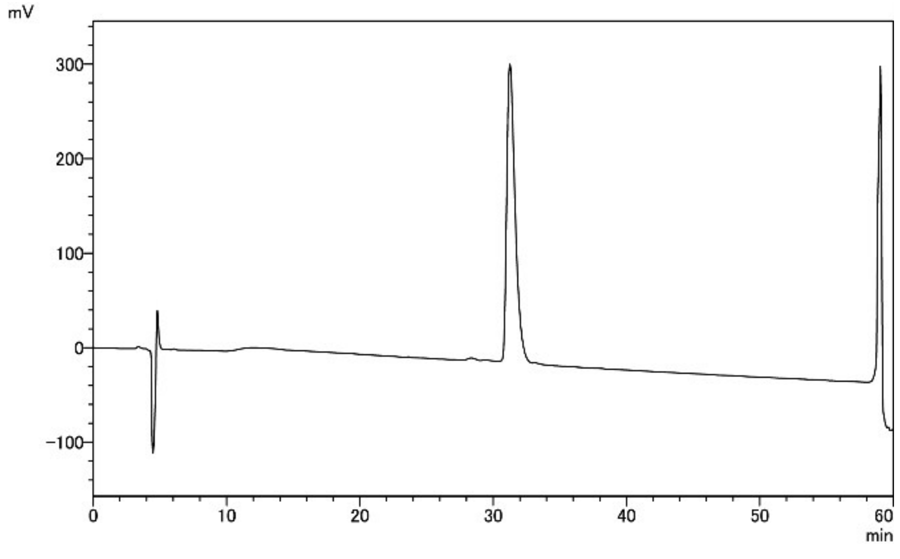


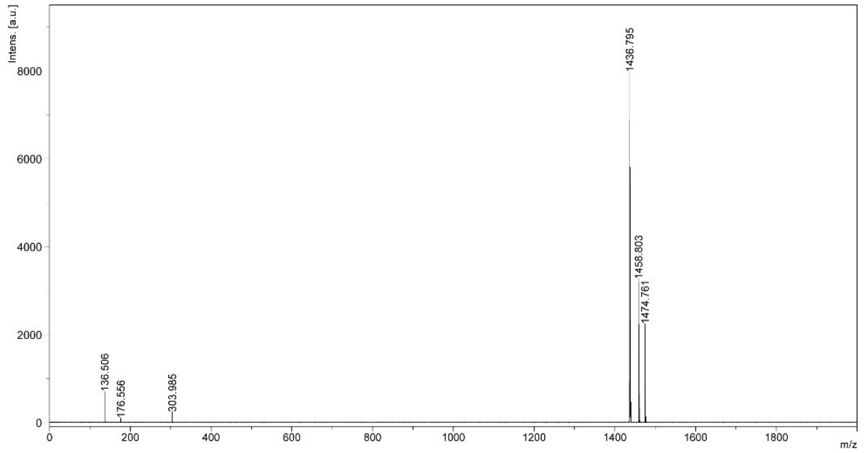

**Figure S13**. Characterization of compound **12**. Analytical HPLC: A : B ($H_2O$ in 0.1%TFA : $CH_3CN$ in 0.1%TFA) A/B gradient 0~50 min, 10~35% B. MALDI-TOFMS: $C_{50}H_{79}N_{13}O_{21}$, theoretical mass: 1197.551, observed mass: 1220.622 $[M+Na]^+$

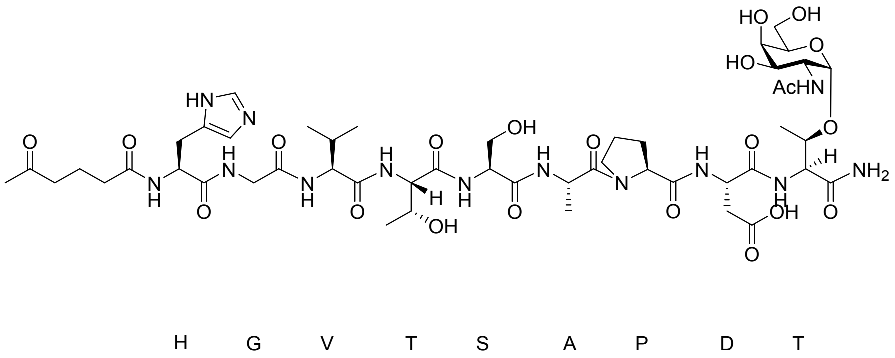


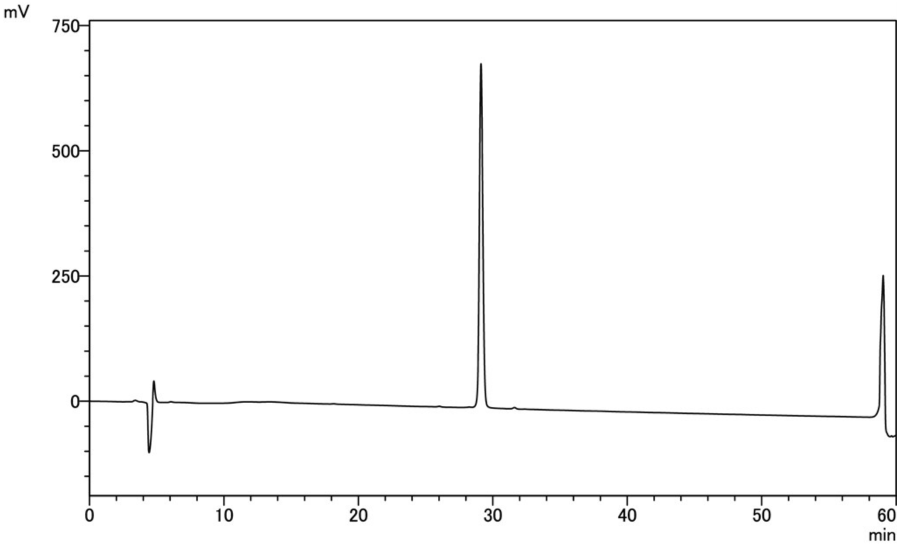


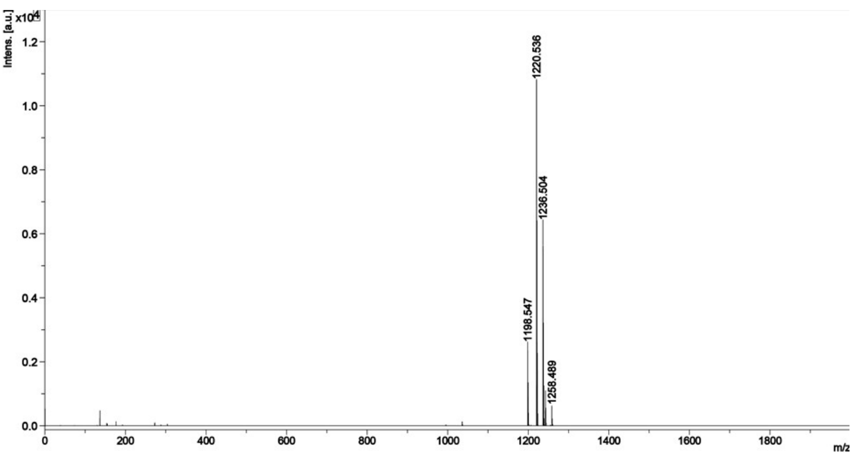

**Figure S14**. Characterization of compound **13**. Analytical HPLC: A : B ($H_2O$ in 0.1%TFA : $CH_3CN$ in 0.1%TFA) A/B gradient 0~50 min, 10~35% B. MALDI-TOFMS: $C_{56}H_{91}N_{17}O_{22}$, theoretical mass: 1353.652, observed mass: 1354.704 $[M+H]^+$

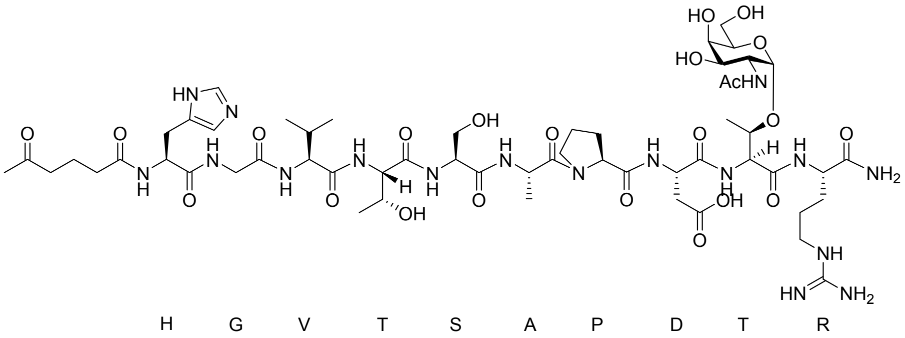


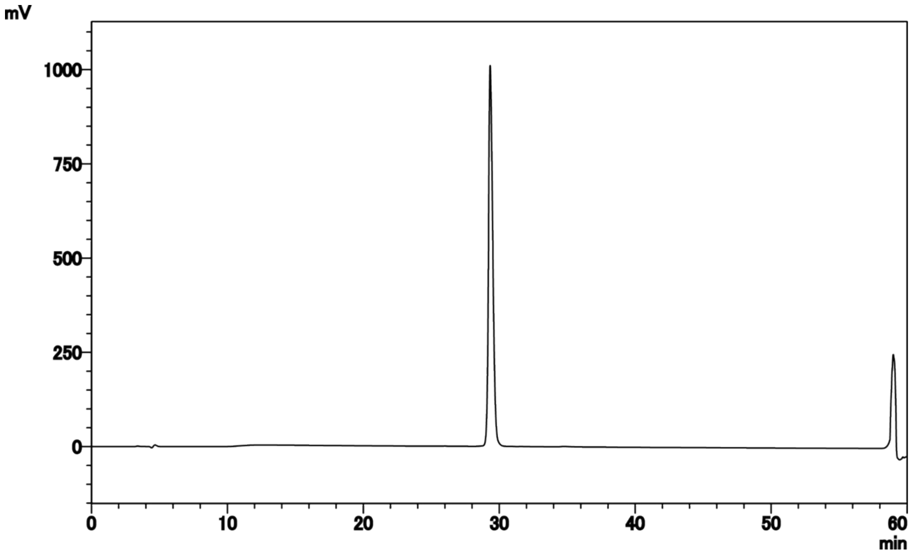


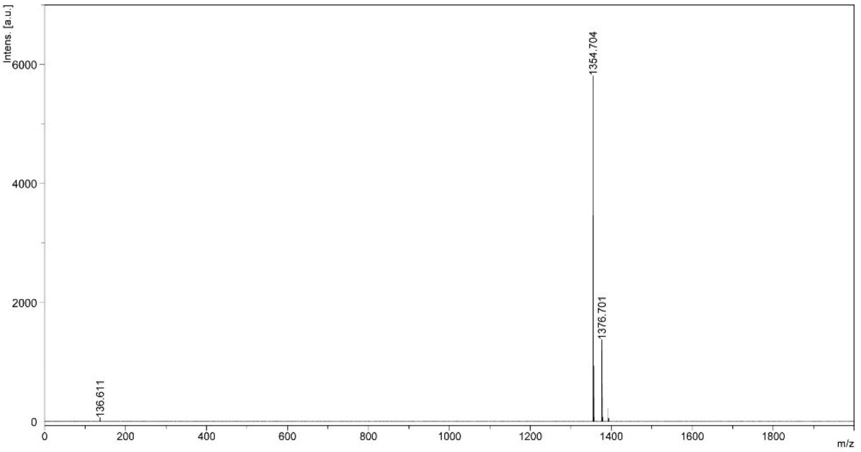

**Figure S15**. Characterization of compound **14**. Analytical HPLC: A : B ($H_2O$ in 0.1%TFA : $CH_3CN$ in 0.1%TFA) A/B gradient 0~50 min, 10~35% B. MALDI-TOFMS: $C_{61}H_{98}N_{18}O_{23}$, theoretical mass: 1450.705, observed mass: 1451.743 $[M+H]^+$

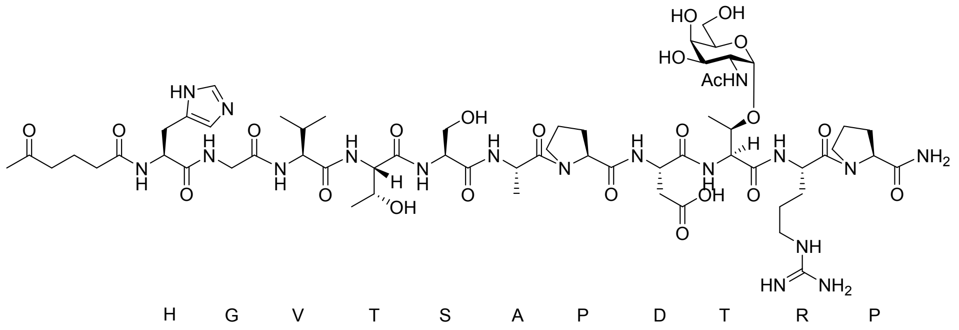


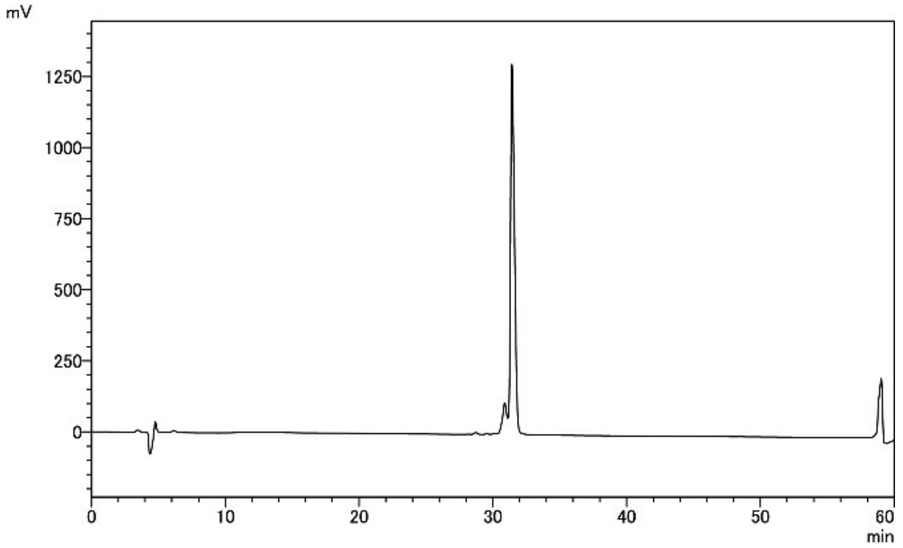


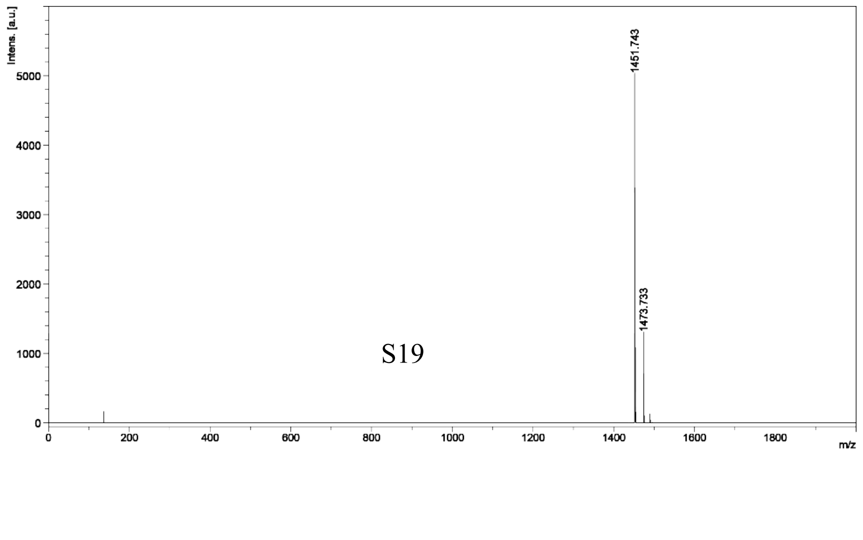

**Figure S16**. Characterization of compound **15**. Analytical HPLC: A : B ($H_2O$ in 0.1%TFA : $CH_3CN$ in 0.1%TFA) A/B gradient 0~50 min, 10~35% B. MALDI-TOFMS: $C_{64}H_{103}N_{19}O_{24}$, theoretical mass: 1521.742, observed mass: 1522.805 $[M+H]^+$

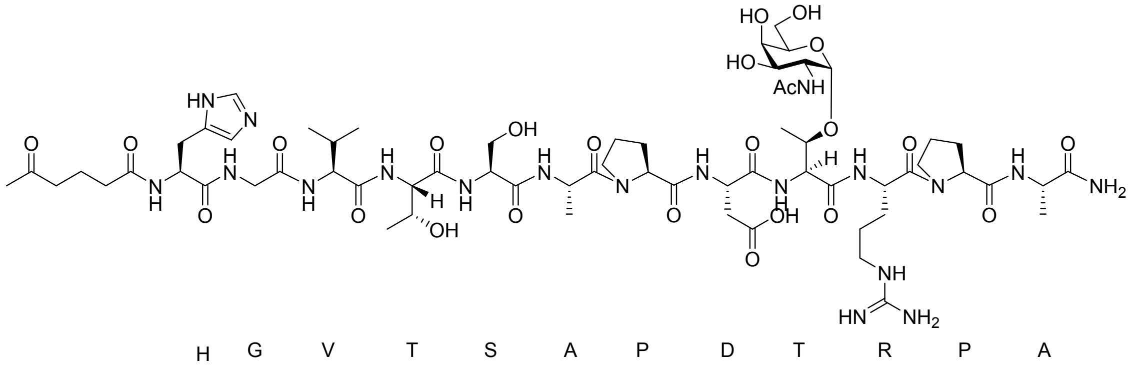


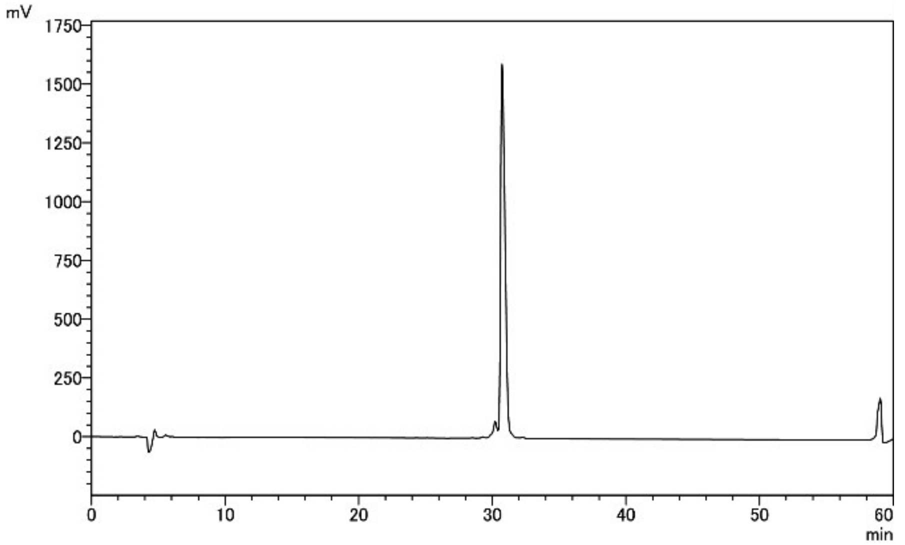


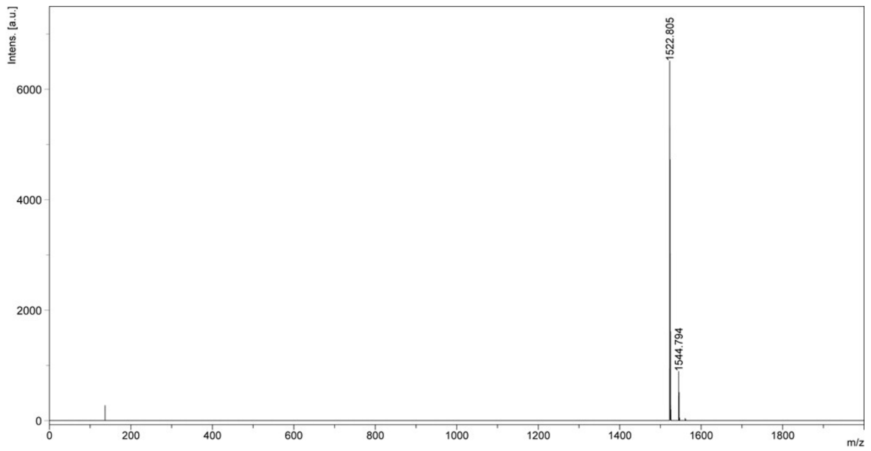

**Figure S17**. Characterization of compound **16**. Analytical HPLC: A : B ($H_2O$ in 0.1%TFA : $CH_3CN$ in 0.1%TFA) A/B gradient 0~50 min, 10~35% B. MALDI-TOFMS: $C_{69}H_{110}N_{20}O_{25}$, theoretical mass: 1618.795, observed mass: 1619.889 $[M+H]^+$

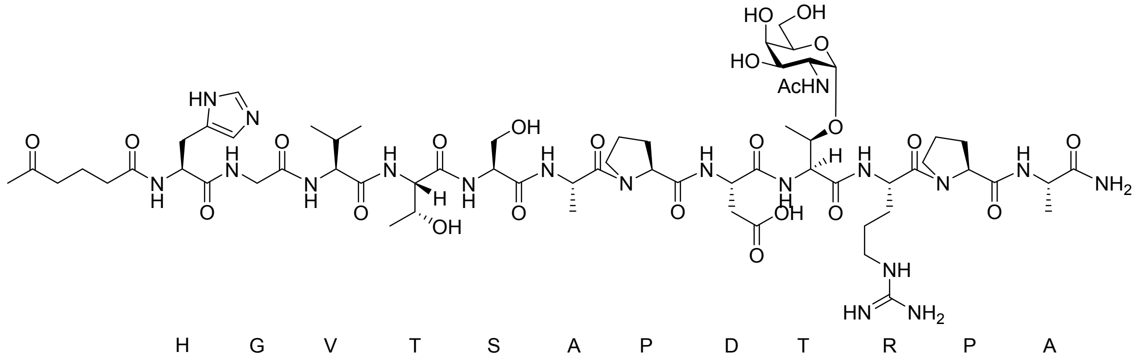


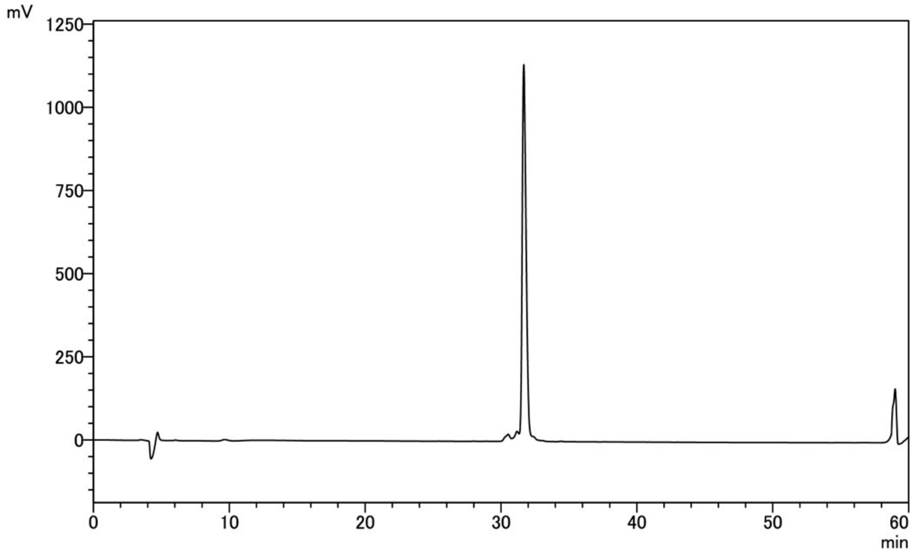


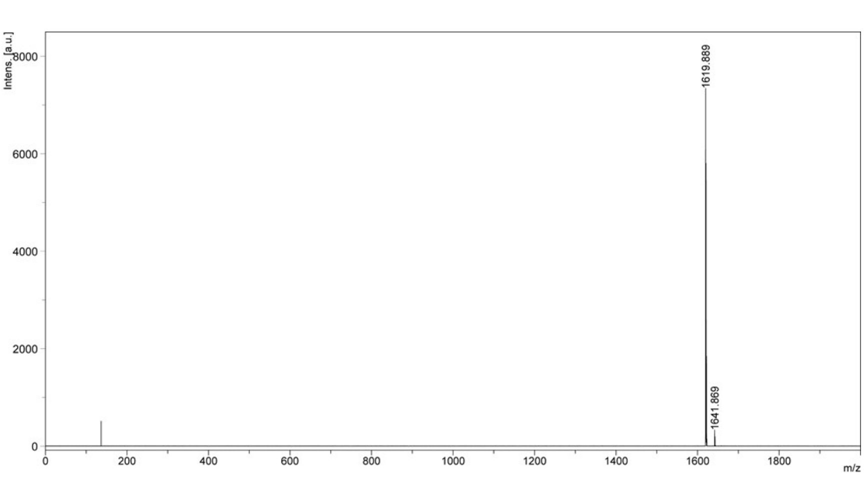

**Figure S18**. Characterization of compound 1**7**. Analytical HPLC: A : B ($H_2O$ in 0.1%TFA : $CH_3CN$ in 0.1%TFA) A/B gradient 0~50 min, 10~35% B. MALDI-TOFMS: $C_{71}H_{113}N_{21}O_{26}$, theoretical mass: 1675.817, observed mass: 1676.889 $[M+H]^+$

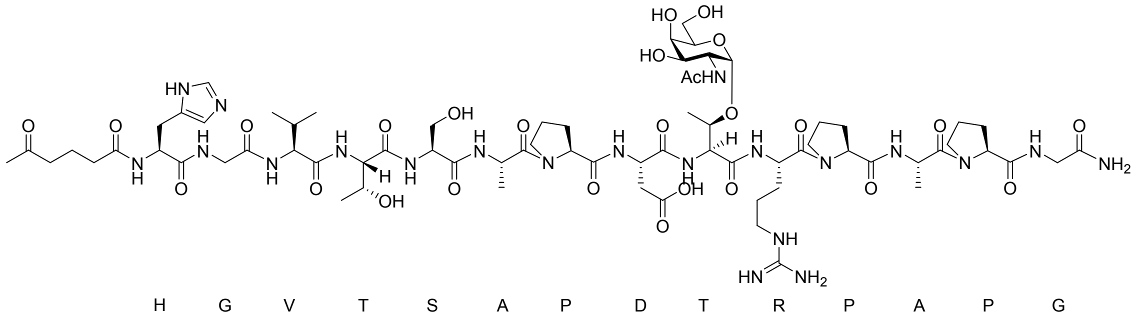


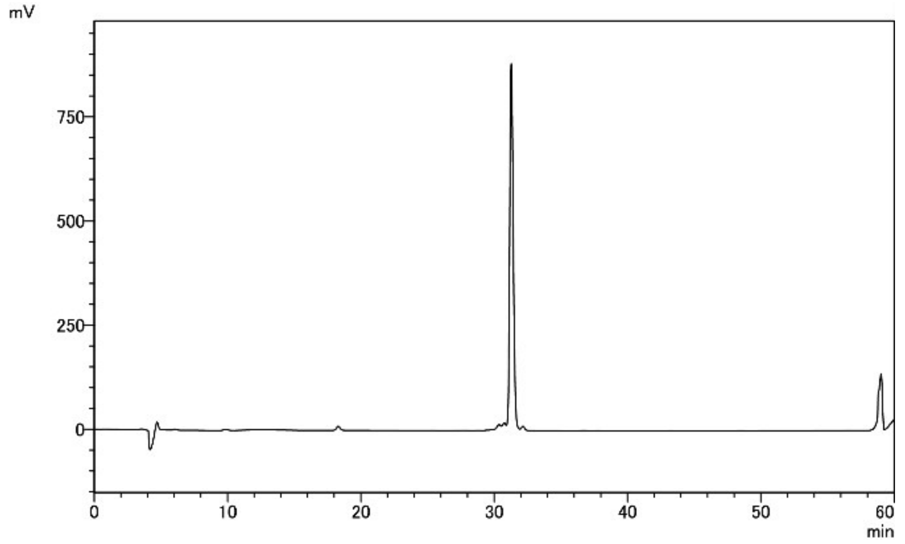


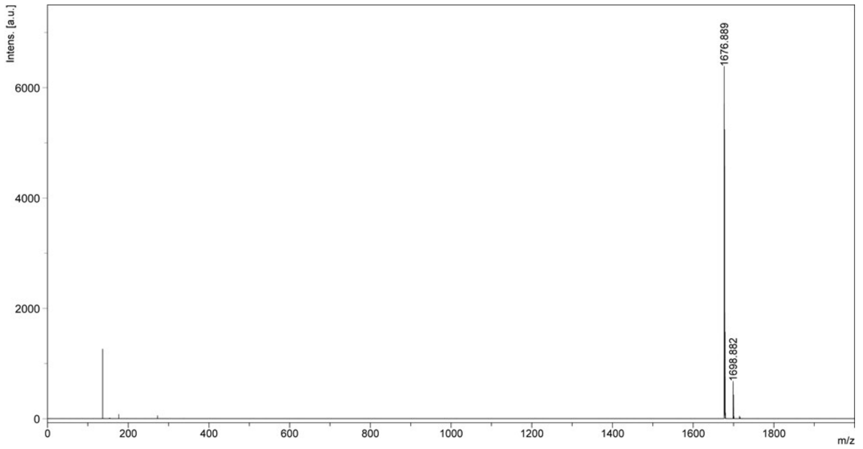

**Figure S19**. Characterization of compound **18**. Analytical HPLC: A : B ($H_2O$ in 0.1%TFA : $CH_3CN$ in 0.1%TFA) A/B gradient 0~50 min, 10~35% B. MALDI-TOFMS: $C_{86}H_{136}N_{26}O_{29}$, theoretical mass: 1996.997, observed mass: 1998.035 $[M+H]^+$

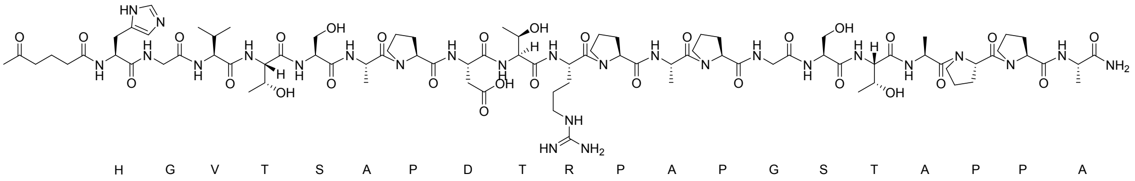


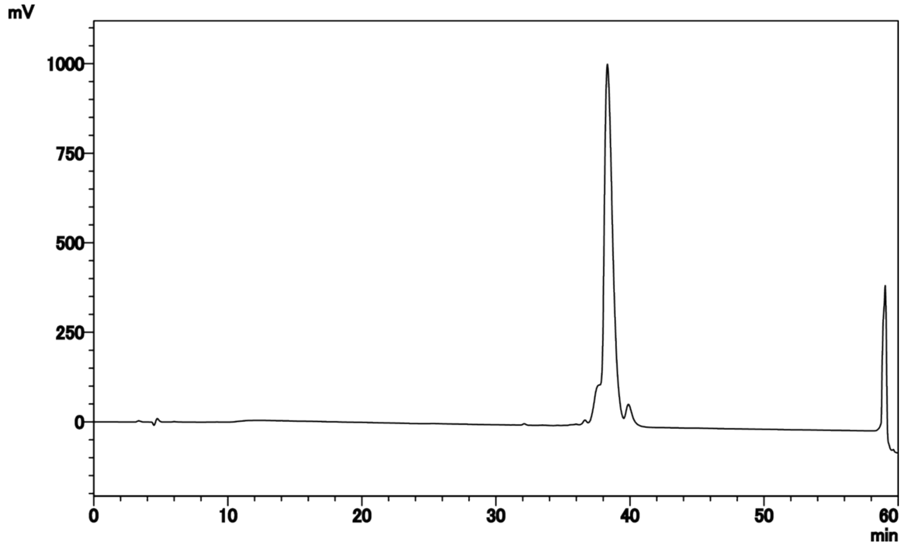


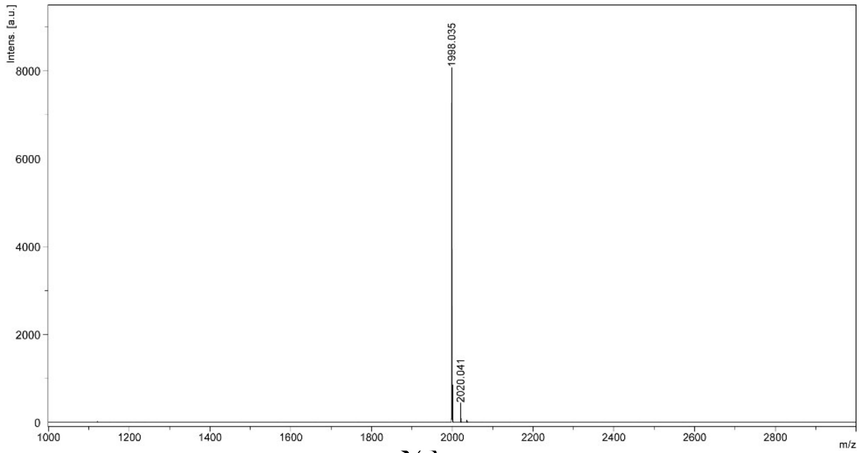

**Figure S20**. Characterization of compound **19**. Analytical HPLC: A : B ($H_2O$ in 0.1%TFA : $CH_3CN$ in 0.1%TFA) A/B gradient 0~50 min, 10~35% B. MALDI-TOFMS: $C_{102}H_{162}N_{28}O_{39}$, theoretical mass: 2403.155, observed mass: 2404.232 $[M+H]^+$

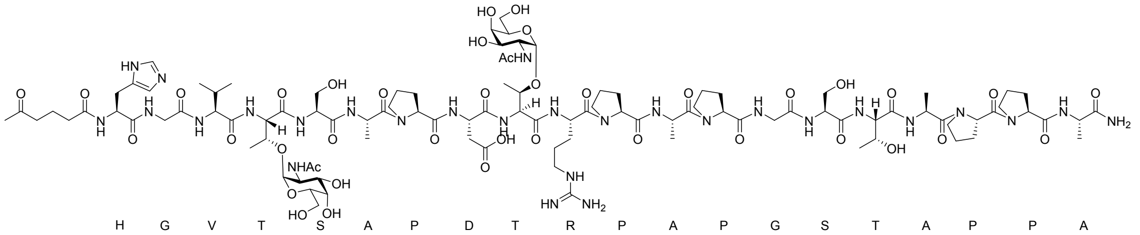


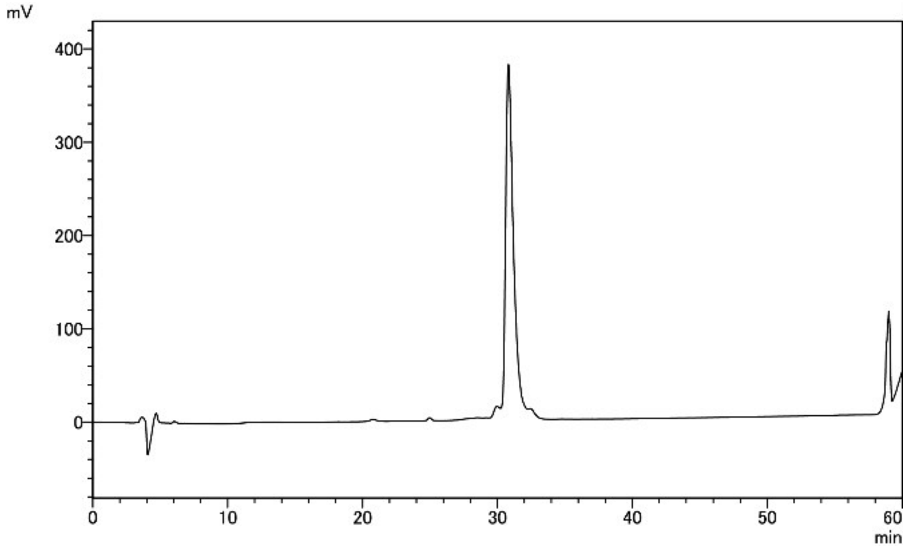


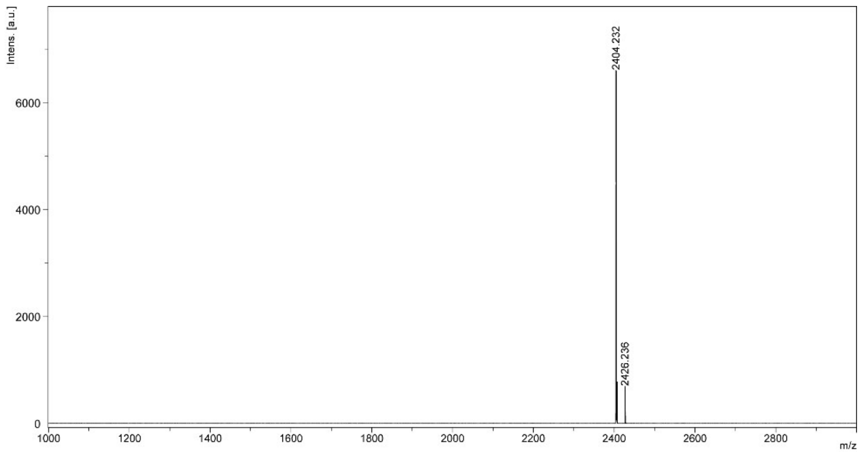

**Figure S21**. Characterization of compound **20**. Analytical HPLC: A : B ($H_2O$ in 0.1%TFA : $CH_3CN$ in 0.1%TFA) A/B gradient 0~50 min, 10~35% B. MALDI-TOFMS: $C_{102}H_{162}N_{28}O_{39}$, theoretical mass: 2403.155, observed mass: 2404.184 $[M+H]^+$

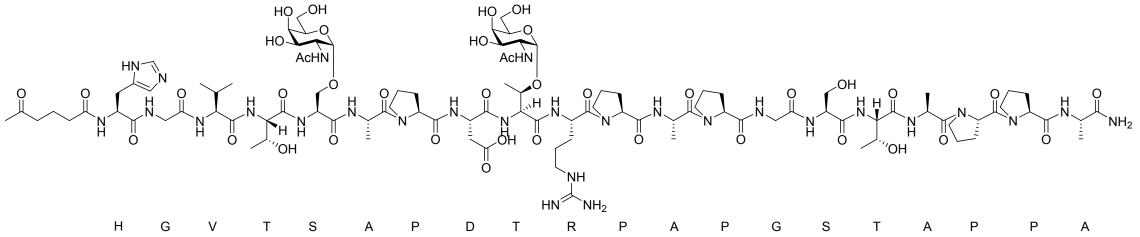


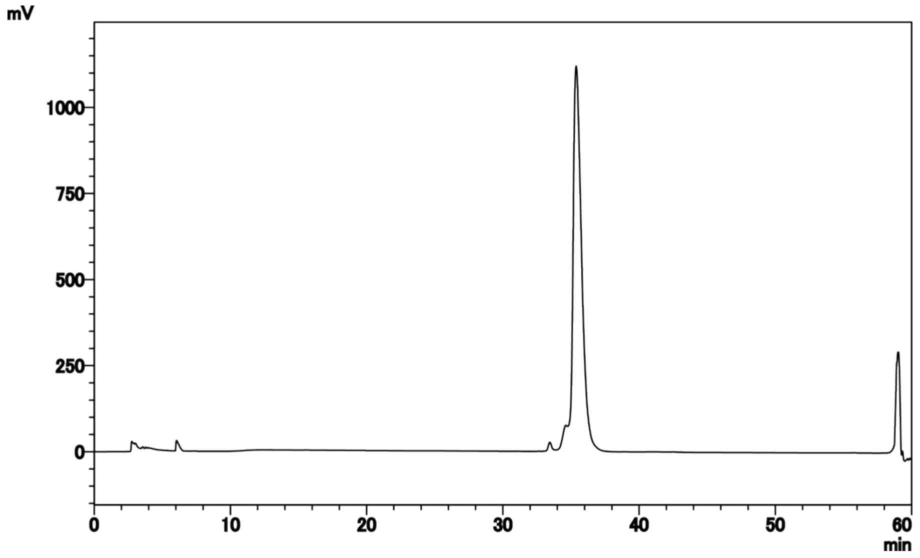


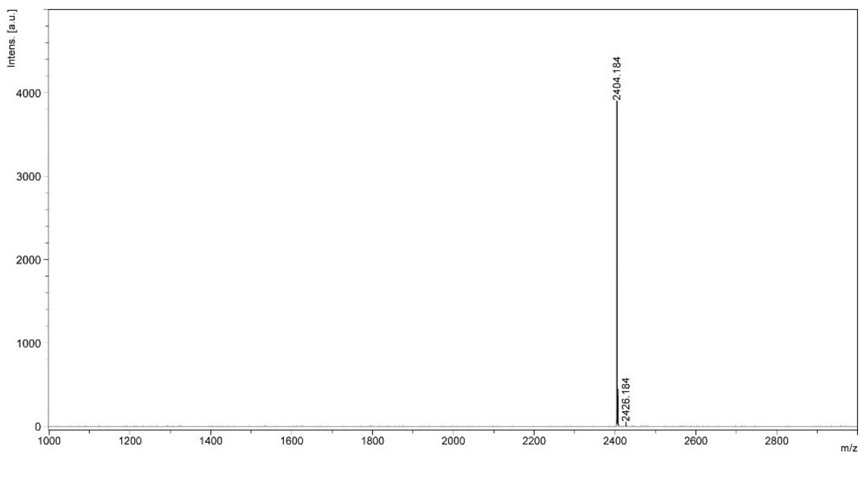

**Figure S22**. Characterization of compound **21**. Analytical HPLC: A : B ($H_2O$ in 0.1%TFA : $CH_3CN$ in 0.1%TFA) A/B gradient 0~50 min, 10~35% B. MALDI-TOFMS: $C_{102}H_{162}N_{28}O_{39}$, theoretical mass: 2403.155, observed mass: 2404.228 $[M+H]^+$

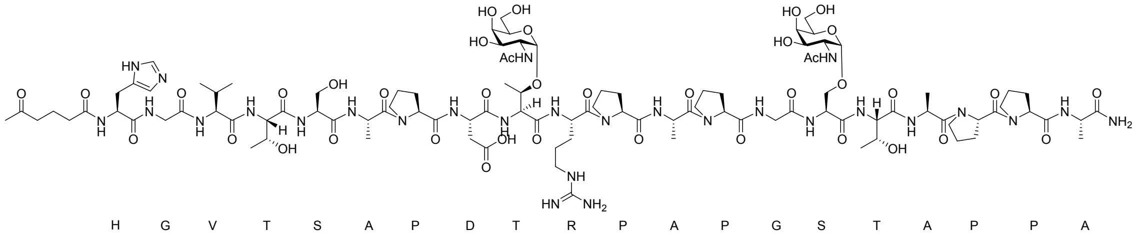


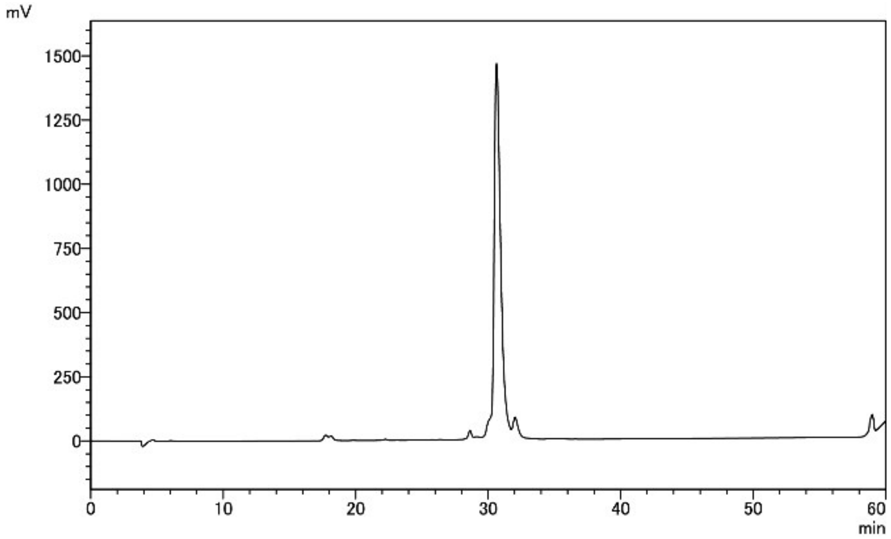


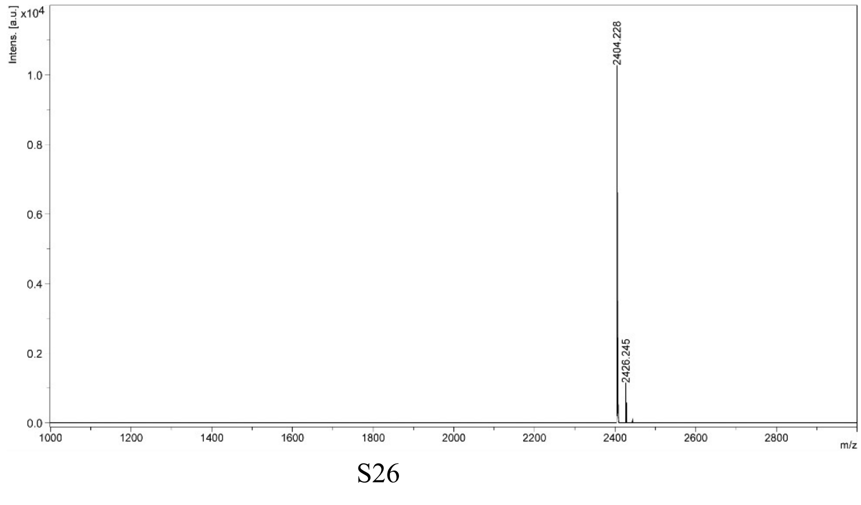

**Figure S23**. Characterization of compound **22**. Analytical HPLC: A : B ($H_2O$ in 0.1%TFA : $CH_3CN$ in 0.1%TFA) A/B gradient 0~50 min, 10~35% B. MALDI-TOFMS: $C_{102}H_{162}N_{28}O_{39}$, theoretical mass: 2403.155, observed mass: 2404.228 $[M+H]^+$

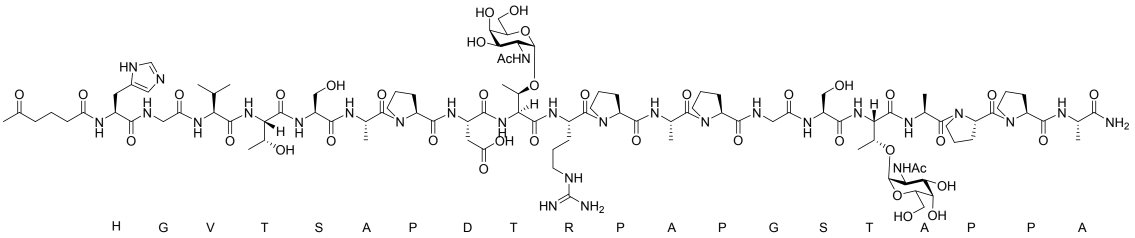


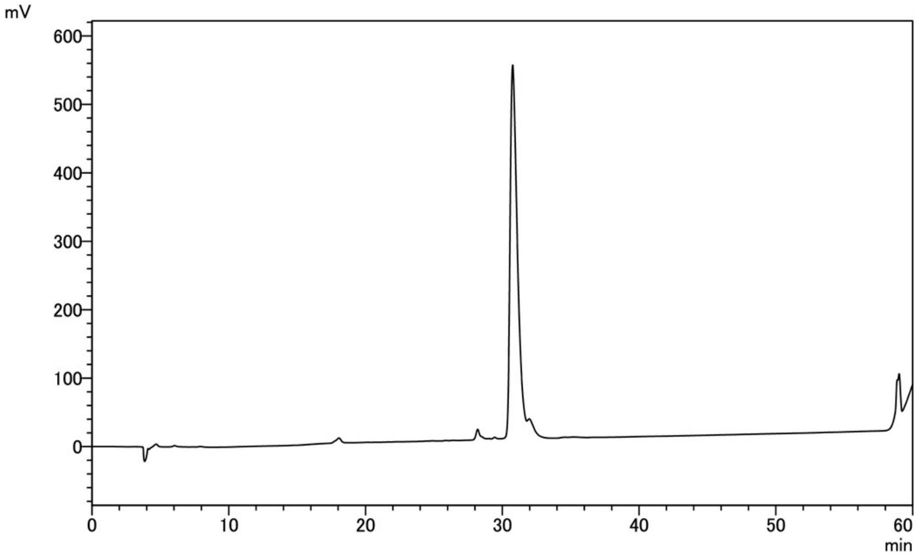


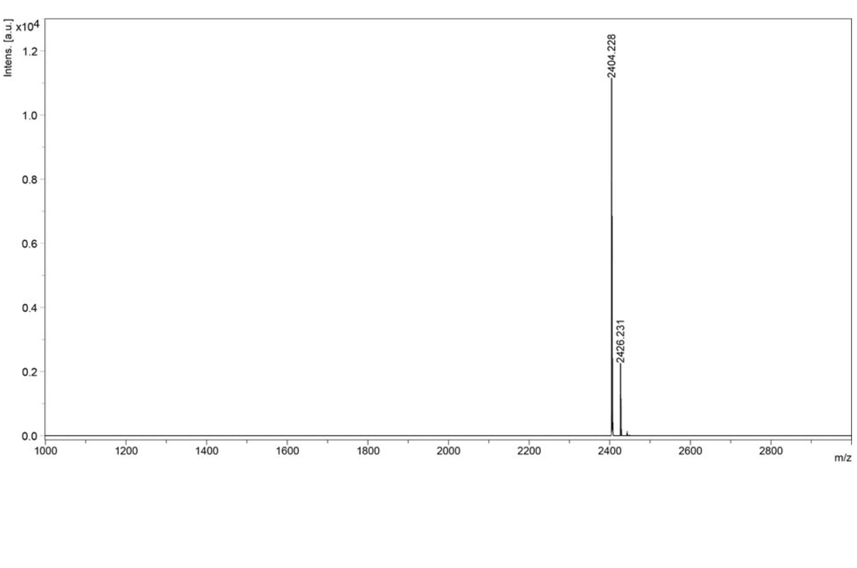

**Figure S24**. Characterization of compound **23**. Analytical HPLC: A : B ($H_2O$ in 0.1%TFA : $CH_3CN$ in 0.1%TFA) A/B gradient 0~50 min, 10~35% B. MALDI-TOFMS: $C_{100}H_{159}N_{27}O_{39}$, theoretical mass: 2362.129, observed mass: 2363.154 $[M+H]^+$

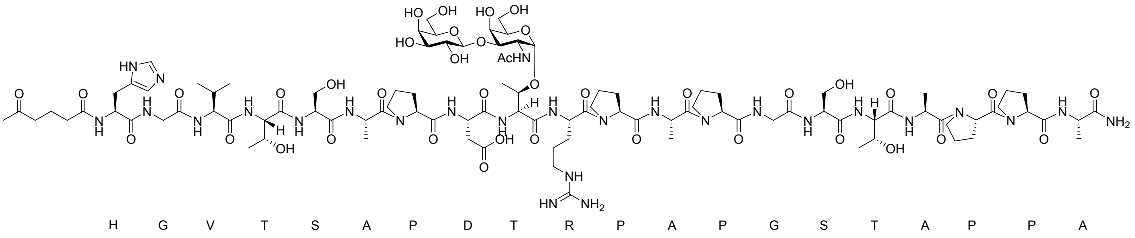


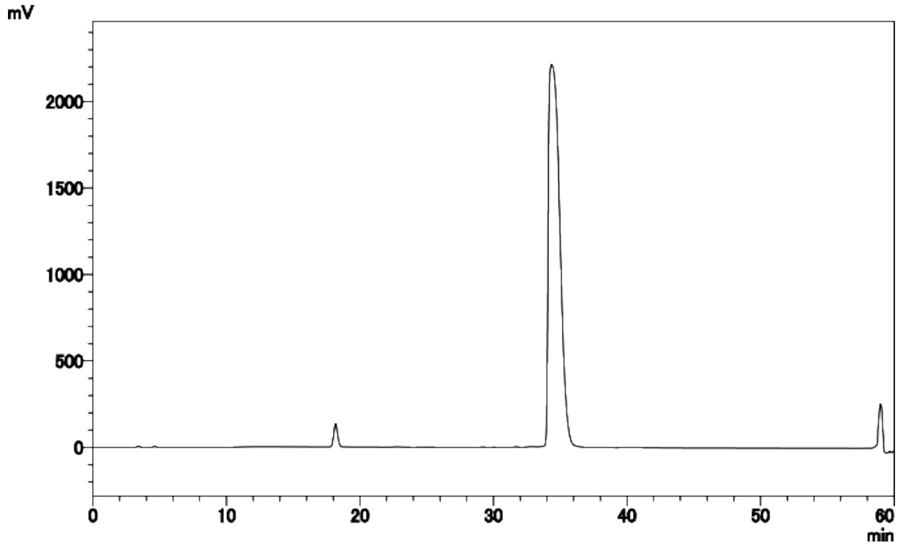


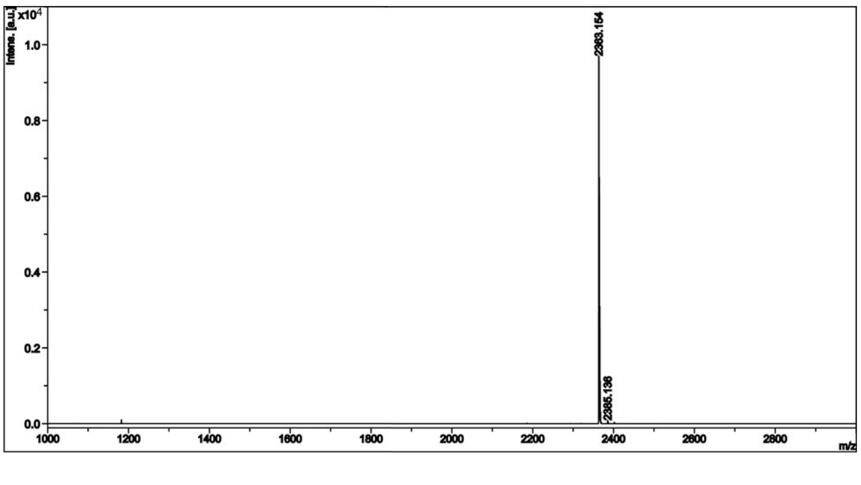

**Figure S25**. Characterization of compound **24**. Analytical HPLC: A : B ($H_2O$ in 0.1%TFA : $CH_3CN$ in 0.1%TFA) A/B gradient 0~50 min, 10~35% B. MALDI-TOFMS: $C_{63}H_{104}N_{20}O_{24}$, theoretical mass: 1670.811, observed mass: 1671.765 $[M+H]^+$

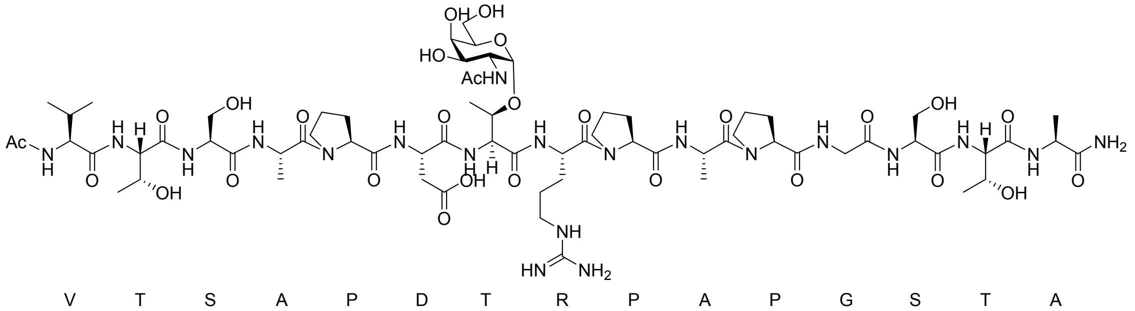


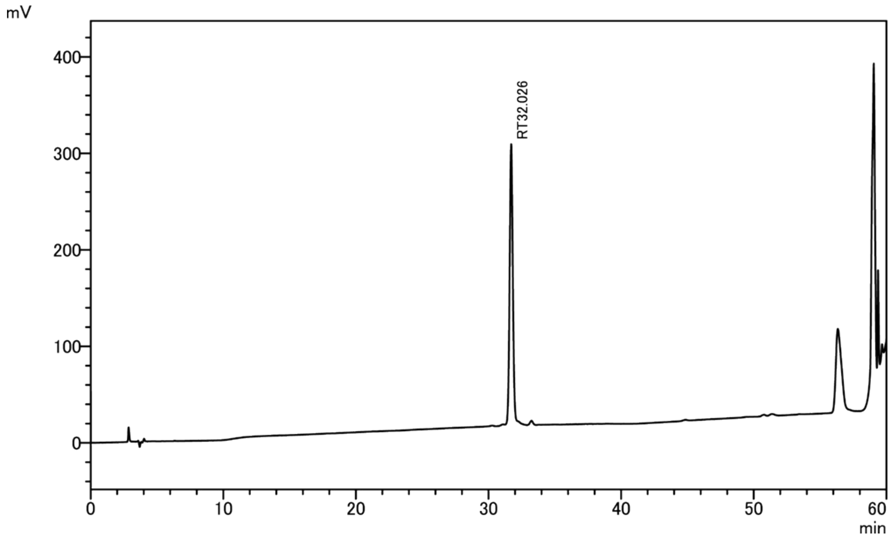


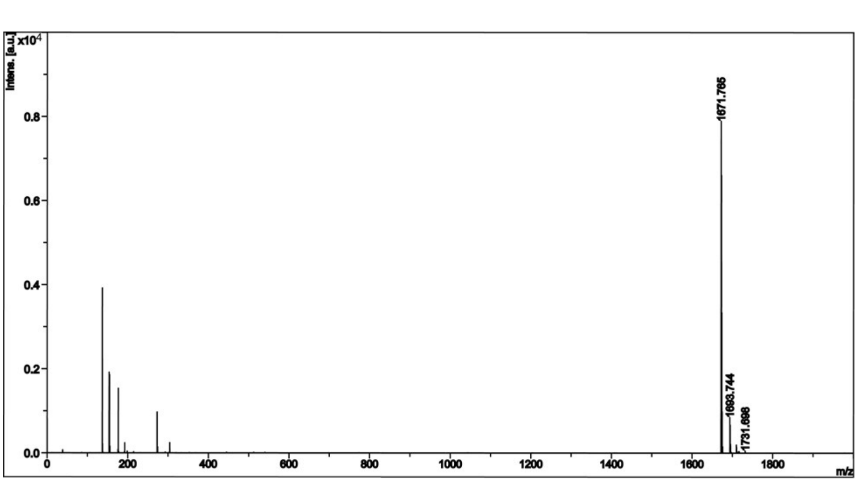

**Figure S26**. Characterization of compound **25**. Analytical HPLC: A : B ($H_2O$ in 0.1%TFA : $CH_3CN$ in 0.1%TFA) A/B gradient 0~50 min, 10~35% B. MALDI-TOFMS: $C_{93}H_{148}N_{28}O_{34}S$, theoretical mass: 2233.043, observed mass: 2234.048 $[M+H]^+$

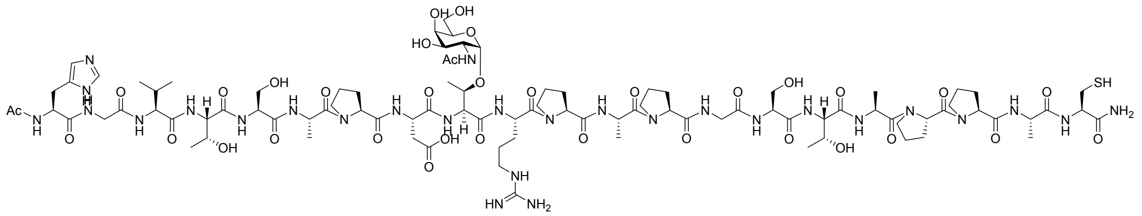


H G V T S A P D T R P A P G S T A P P A C

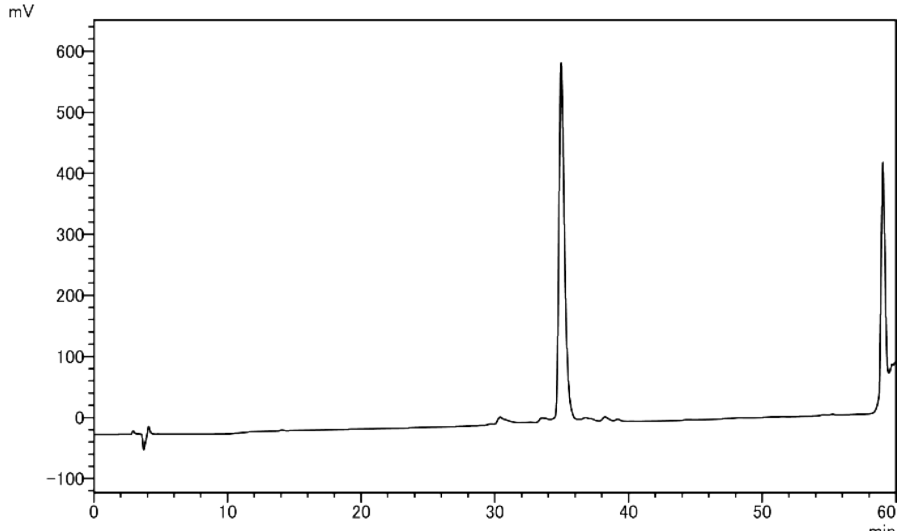


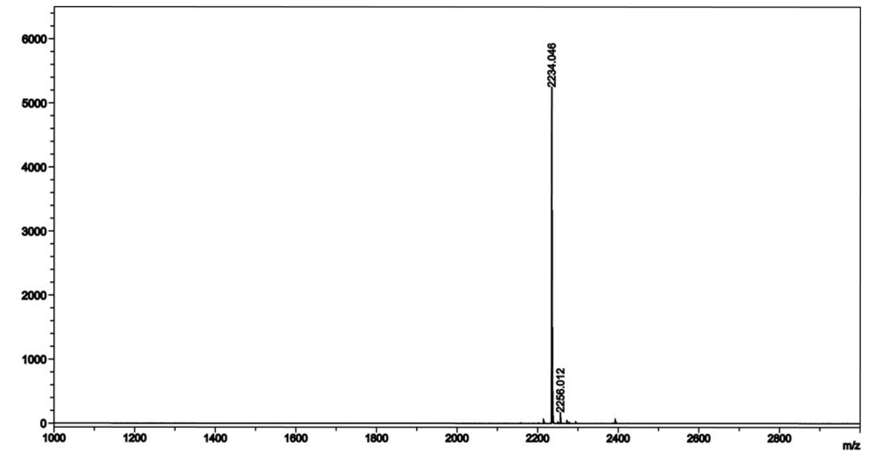

**Supplementary references**


S1. Tachibana, Y. et al., Antifreeze glycoproteins: Elucidation of the structural motifs that are essential for antifreeze activity. *Angew. Chem., Int. Ed*. 2004, **43**, 856-862.

S2. Matsushita, T., et al., Rapid microwave-assisted solid-phase glycopeptide synthesis. *Org. Lett*. 2005, **7**, 877–880.

S3. Ohyabu, N. et al., An essential epitope of anti-MUC1 monoclonal antibody KL-6 revealed by focused glycopeptide library. *J. Am. Chem. Soc*. 2009, **131**, 17102-17109.

S4. Sado, Y. et al., Lymphocytes from enlarged iliac lymph nodes as fusion partners for the production of monoclonal antibodies after a single tail base immunization attempt. *Acta Histochem. Cytochem*. 2006, **39**, 89-94.

S5. Hoogenraad, N. et al., The effect of pre-injection of mice with pristane on ascites tumour formation and monoclonal antibody production. *J. Immunol. Methods* 1983, **61**, 317-320.

S6. Mariuzza, R. A. et al., Preliminary crystallographic study of the complex between the Fab fragment of a monoclonal anti-lysozyme antibody and its antigen. *J. Mol. Biol.* 1983**, 170**, 1055-1058.

S7. Belenky, A. S. et al. Absence of protein G-Fc interaction in ficin-derived mouse IgG1 digests. *J. Immunoass. Immunochem.* 2003, **24**, 311–318.

S8. Kabsch, W. Xds. *Acta Crystallogr. Sect. D Biol. Crystallogr.* 2010, **66**, 125–132.

S9. McCoy, A. J. et al. Phaser crystallographic software. *J. Appl. Crystallogr.* 2007**, 40**, 658–674.

S10. Adams, P. D. et al. PHENIX: A comprehensive Python-based system for macromolecular structure solution. *Acta Crystallogr. Sect. D Biol. Crystallogr.* 2010**,** **66**, 213–221.

S11. Emsley P.; Cowtan, K. Coot: Model-building tools for molecular graphics. *Acta Crystallogr. Sect. D Biol. Crystallogr.* 2004, **60**, 2126–2132.

S12. Chen, V. B. *et al.* MolProbity: All-atom structure validation for macromolecular crystallography. *Acta Crystallogr. Sect. D Biol. Crystallogr.* 2010, **66**, 12–21.

S13. Matsushita, T. et al. A straightforward protocol for the preparation of high performance microarray displaying synthetic MUC1 glycopeptides. *Biochim. Biophys. Acta - Gen. Subj.* 2014, **1840**, 1105–1116.

S14. Artigas, G. et al., Synthetic Mucin-Like Glycopeptides as Versatile Tools to Measure Effects of Glycan Structure/Density/Position on the Interaction with Adhesion/Growth-Regulatory Galectins in Arrays. *Chem. Asian J.* 2017, **12**, 159–167.
